# The Photosynthetic Complexes of Thylakoid Membranes of Photoautotrophs and a Quartet of Their Polar Lipids

**DOI:** 10.3390/ijms26209869

**Published:** 2025-10-10

**Authors:** Anatoly Zhukov, Vadim Volkov

**Affiliations:** K.A. Timiryazev Institute of Plant Physiology, Russian Academy of Sciences, Botanicheskaya Street 35, Moscow 127276, Russia

**Keywords:** fatty acids, lipids, mono- and digalactosyldiacylglycerols, phosphatidylglycerols, photosynthesis, sulfoquinovosyldiacylglycerols, thylakoid membrane

## Abstract

The important function of polar lipids in the biochemical chains of photosynthesis, the outstanding biochemical process on our planet, has been mentioned in many publications. Over the last several years, apart from the known function of lipids in creating a matrix for photosynthetic complexes, most attention has been paid to the role of lipids in building up and functioning of the photosynthetic complexes. The lipid molecules are found inside the complexes of photosystem II (PSII), photosystem I (PSI), and cytochrome b_6_f (Cyt b_6_f) together with other cofactors that accompany proteins and chlorophyll molecules. Super complexes PSII-light-harvesting complex II (PSII-LHCII) and PSI-light-harvesting complex I (PSI-LHCI) also include lipid molecules; part of the lipid molecules is located at the borders between the separate monomers of the complexes. Our interest is in the exact localization of lipid molecules inside the monomers: what are the protein subunits with the lipid molecules in between and how do the lipids contact directly with the amino acids of the proteins? The photosystems include very few classes of all the polar lipids, three groups of glyceroglycolipids, and one group of glycerophospholipids make up the quartet of polar lipids. What are the reasons they have been selected for the role? There are no doubts that the polar heads and the fatty acids chains of these lipids are taking part in the processes of photosynthesis. However, what are the distinct roles for each of them? The advantages and disadvantages of the head groups of lipids from thylakoid membranes and those lipids that for various reasons could not take their place are discussed. Attention is focused on those bound fatty acids that predominate or are characteristic for each class of thylakoid lipids. Emphasis is also placed on the content of each of the four lipids in all photosynthetic complexes, as well as on contacts of head groups and acyl chains of lipids with specific proteins, transmembrane chains, and their amino acids. This article is devoted to the search for answers to the questions posed.

## 1. Introduction

Oxygenic photosynthesis is a fundamental biological process that forms the basis of aerobic life on our planet. This process, carried out by cyanobacteria, algae, and higher plants, uses light energy from the Sun to convert water and carbon dioxide into sugars with the release of molecular oxygen. Thus, the photosynthetic splitting of water can be considered as one of the most important of all biochemical reactions, e.g., [1,2,3,4]. At the same time, the efficient absorption of light and the conversion of its energy by chlorophyll (Chl) are based on the high degree of organization of internal membrane structures of chloroplasts.

Chloroplasts possess inner and outer membranes and also the internal intensively developed membranous system of thylakoid membranes. Photosynthetic photochemical reactions and electron transfer are realized in this thylakoid membrane which is a bilayer formed by glycerolipids with thousands of embedded proteins, pigments and photosynthetic cofactors. Chloroplasts presumably have cyanobacterial origin [5], therefore the lipid composition of the thylakoid membranes is similar for chloroplasts and cyanobacteria [6,7]. In terms of lipid and fatty acid (FA) composition, thylakoid membranes, which are typically enriched in several types of glycerophospholipids, differ from other cellular membranes [6].

In oxygenic photosynthesis, four protein cofactor complexes, namely photosystem II (PSII), cytochrome b_6_f (Cyt b_6_f), photosystem I (PSI), and ATP synthase are embedded in the lipid bilayer of thylakoid membranes of various organisms forming nanodomains, e.g., [8] like lipid rafts, e.g., [9]. They are responsible for the capture and conversion of light energy to ATP and nicotinamide adenine dinucleotide phosphate (NADP^+^) and then to its reduced form (NADPH) for further carbon dioxide fixation. In cyanobacteria and chloroplasts of plants and green algae, photosynthetic reactions occur within the thylakoid membrane, where three major complexes—PSII, Cyt b_6_f and PSI—form an electron transport chain together with plastoquinones and plastocyanins, which carry free electrons [2,10,11]. In this case, the reactions of light energy conversion are realized in PSII and PSI, both of which are huge multi-subunit pigment–protein complexes [1,3,4,12,13,14,15]. In addition to the main complexes, in chloroplasts of some plant species and in cyanobacteria NDH or NDH-1 complex (NADH dehydrogenase or type I NADH dehydrogenase) is found to provide cyclic electron flow around PSI; this electron flow is connected to chlororespiration, acclimation to environment, hydrogen production and CO_2_-concentration mechanisms in different organisms [16,17,18,19].

Each of the two supramolecular machines, PSII and PSI, consists of a core complex (reaction center or core) and a peripheral antenna system. The reaction centers (RC) of PSII and PSI, where photosynthetic charge separation occurs, consist of several protein subunits and cofactors, including chlorophylls, carotenoids, quinones, and lipids [2,4]. The antenna systems of PSII and PSI, which serve to capture light energy and efficiently transfer it to RCs of photosystems, consist of the major light-harvesting complexes (LHCII and LHCI) [4,20].

**Figure 1:**
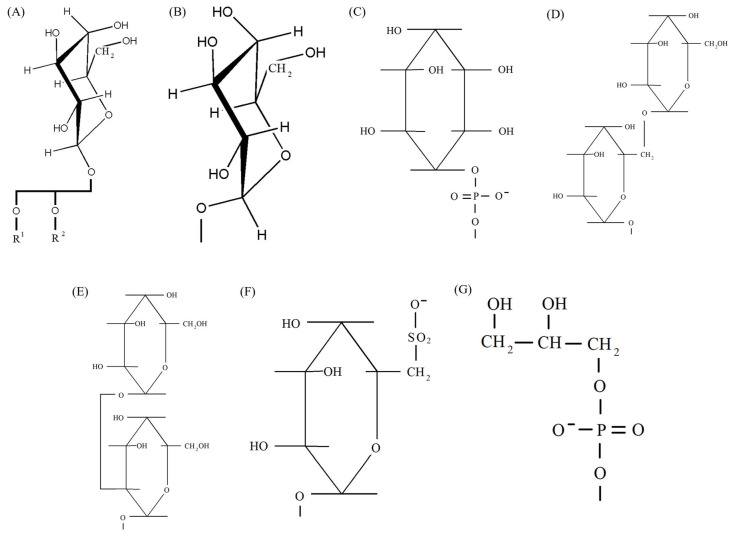
Head groups of lipids and their possible substituents that function in photosystems. (**A**) β-galactopyranosyl of MGDG with schematically depicted remaining part of the lipids; (**B**) α-glucopyranosyl of MGlcDAG; (**C**) myoinositol of PI; (**D**) α-galactopyranosyl-β-galactopyranosyl of DGDG; (**E**) α-galactopyranosyl-α-glucopyranosyl of galactosyl glucosyl-DG; (**F**) deoxysulfo-α-glucose of SQDG; (**G**) glycerol and phosphate of PG. (**A**,**B**) were generated using ChemDraw Pro 8.0.

A common property of thylakoid membranes of cyanobacteria, algae and plants is the creation of a hydrophobic liquid matrix where photosynthetic complexes are embedded, as well as the formation of a barrier to the free diffusion of protons and other ions through these membranes. This is a necessary condition to develop electrochemical potential difference at the membrane, which stimulates ATPase, and to form a strong reducing agent capable of reducing NADP^+^ [11,13,20,21,22]. Such a matrix is created by glycerolipid bilayers, which consist of three unique classes of glyceroglycolipids and only one class of glycerophospholipids (Figure 1) (with a few minor inclusions of other lipids discovered in several species where additional studies are needed).

In addition, X-ray crystallography (XRC) and single-particle cryo-electron microscopy (cryo-EM) have revealed molecules of these glycerolipids directly in the structure of PSII, PSI, Cyt b_6_f, and NDH-1 complexes [18]. For example, each monomer of PSII-LHCII complex of *Spinacia oleracea* contains 34 lipid molecules in addition to 105 Chl molecules [23,24]. In the future, new results can be expected from the application of multiscale molecular dynamics simulations [25,26,27,28]. Thus, lipids play an important role in the functioning of photosystems, not only by forming a membrane lipid matrix for them, but also by contributing directly to their structure [6,10,13,20,23,29,30]. Further studies are needed to clarify the role of individual lipid molecules associated with specific sites in photosynthetic complexes, both for assembly of these complexes and for the processes of photosynthesis in general. The aim of the review is to briefly introduce the supramolecular complexes of photosynthetic thylakoid membranes, to stress the few known roles of lipids in their functioning, and to suggest future potential directions of research. This review adds to the previous comprehensive reviews of 2007 and 2002 on lipids in photosynthetic reaction centers [20,25], but with a wider scope of emphasizing the best novel results and long-term questions from experimental research on plant lipids over several decades.

## 2. Thylakoid Membranes

**Figure 2:**
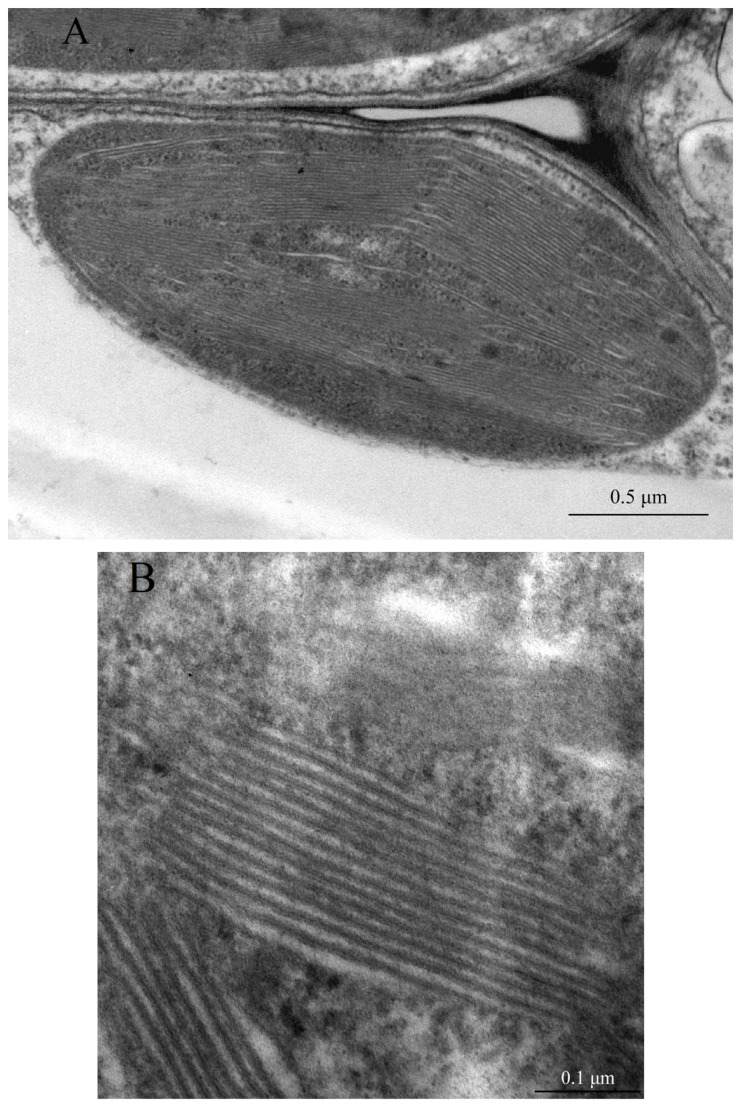
Plant developed photosynthesizing chloroplasts are filled with a network of thylakoid membranes organized in free lamellae with stacked grana. (**A**) Representative chloroplast of a tomato plant *Solanum lycopersicum* L. grown under intensive illumination; (**B**) higher resolution image demonstrates better details of the organization of grana and free lamellae in the stroma of these tomato chloroplasts. The electron microscopic images are courtesy of Lyudmila Khalilova, K.A. Timiryazev Institute of Plant Physiology, Russian Academy of Sciences.

The inner region of chloroplasts, the stroma, contains an extensive united membrane network, the thylakoid membranes, in which the processes of photosynthesis are localized. These thylakoid membranes, or thylakoids, are collected into lamellae, most of which form stacks called grana and which are connected to each other by free lamellae of the stromal thylakoids (Figure 2). Protein complexes of the photosystems are distributed between the lamellae of the grana and the free lamellae of the thylakoids. Most of PSII and LHCII are predominantly located inside the lamellae of the grana, while PSI, together with LHCI and ATP synthetase, mainly function in the unpacked lamellae of the stroma and at the marginal regions of the grana [4,21,31,32,33,34,35] (Figure 2). A small portion of PSII is also present in the unpacked lamellae of the stroma. The LHCII antenna complexes surround the PSII core complex, absorb light energy and transfer it to the reaction centers of photosystems (RC), causing charge separation in a special Chl pair called P680. The interaction of LHCII with monogalactosyldiacylglycerols (MGDG) maintains an optimal lipid: protein ratio, which is important not only during the processes of thylakoid membrane development and maturation, but also for maintaining tight protein packing and regulating the flexibility and stability of structures in the grana region [21]. Cyt b_6_f is found in both folded and unfolded membranes [24,36]. Differentiation into granular and stromal membranes depends on the interaction of various processes. Tight packing of thylakoid membranes in granal stacks is due to van der Waals interactions and cation-mediated electrostatic interactions between proteins in opposite membranes [14,15,32]. The thickness of the thylakoid membrane reversibly changes under illumination from around 4.3 to 3.2 nm [37].

The first reaction that occurs in oxygenic photosynthesis is the splitting of water into electrons, protons, and molecular oxygen, of which the electrons and protons are used to synthesize ATP and NADPH, while the oxygen is released into the atmosphere. The water splitting reaction is catalyzed by PSII, which is present in all phototrophs from prokaryotic cyanobacteria to higher plants [3,12,38,39].

The light reactions of photosynthesis in cyanobacteria, eukaryotic algae, and vascular plants depend on the functioning of PSII, Cyt b_6_f, and PSI, which act in a sequence. Driven by photon energy, the light reaction mechanisms perform a linear transfer of electrons from water to ferredoxin/flavodoxin to regenerate NADP^+^. During the process, protons accumulate in the lumen of a thylakoid: the gradient of these protons generated across the membrane is used by ATPase to synthesize ATP [6,12,40,41].

To summarize, PSII oxidizes water to form oxygen while PSI produces NADPH through its electron acceptor ferredoxin and also performs the functions for further ATP production [12,41]. Both PSII and PSI are highly conserved and exist in different oligomeric states in photosynthetic membranes of different species. As a rule, PSII acts exclusively as a dimer while PSI functions as a monomer in chloroplasts of land plants or as an oligomer in most cyanobacteria [1,12,15,41,42,43,44].

Glycerolipids, along with proteins and photosynthetic pigments, are the main and essential components of thylakoid membrane [11,20]. Oxygenic photosynthesis in cyanobacteria and chloroplasts requires a unique lipid composition that is conserved in all oxygenic phototrophs, from cyanobacteria and algae to modern higher plants. Naturally selected glycerolipids function as structural components of several photosystems in the thylakoid membrane and participate directly or indirectly in photosynthetic reactions [6,13].

From the point of their spatial localization, lipids of thylakoid membranes could be divided into three distinct groups: (1) structural lipids that form lipid bilayers; (2) circular/surrounding lipids that are located around protein-cofactor supercomplexes and interact with their outer surface; and (3) integral lipids which are a part of protein-cofactor supercomplexes, reside at interfaces of protein subunits within the supercomplexes and perform essential functions in folding and assembly of protein subunits [21,29,45].

Membrane glycerolipids can be classified according to their physical properties in aqueous dispersions as non-bilayer and bilayer lipids. The geometry of lipid molecules is determined by the size of their head group, degree of unsaturation of alkyl chains, and the individual characteristics of these chains. The lipid composition of all thylakoid membranes is made of four glycerolipids: monogalactosyldiacylglycerols (MGDG), digalactosyldiacylglycerols (DGDG), sulfoquinovosyldiacylglycerols (SQDG), and phosphatidylglycerols (PG) [6,11].

MGDG and DGDG, the major lipids of thylakoid membranes, are the most abundant classes of lipids on Earth. However, the role of the high content of these lipids in thylakoids has been poorly studied [46]. MGDG are cone-shaped and consist of a relatively small head group of a single galactose moiety (Figure 1A) and an extended tail of polyunsaturated FAs. This geometry allows MGDG to form a hexagonal phase II, where its polar head group faces the center of the micellar tubules forming the phase. Being non-bilayer and cone-shaped, MGDG creates tension in the lipid matrix, which promotes the formation of lamellar structures. The presence of wedge-shaped MGDG molecules is important for the formation of membrane curvatures and thylakoid formation. Possible contributions of MGDG include promotion of spontaneous curvature, reduction in excess membrane area, and specific tasks for protein functionality [11,20,23,30,46,47,48]. In addition, both MGDG and DGDG head groups are capable of forming numerous hydrogen bonds with each other and with other compounds [49].

Low-curvature lipids such as DGDG, SQDG, PG, phosphatidylcholines (PC), and phosphatidylinositols (PI) have a relatively cylindrical shape. When mixed with water, they form lamellar bilayer phases corresponding to a classical bilayer. High-negative-curvature lipids—MGDG, diphosphatidylglycerols (DPG), phosphatidylethanolamines (PE), and phosphatidylethanolserines (PS) tend to form the HII phase (inverted hexagonal) or a cubic phase. MGDG and DGDG are non-ionic lipids and function primarily as structural lipids for the formation of lipid bilayers. They provide an amphipathic membrane environment for photosynthetic complexes [23,29,46]. At the same time, SQDG and PG are negatively charged (acidic) lipids and are probably necessary for maintaining the balance of such charges in thylakoid membranes (Figure 1). Also of note, in prokaryotic cyanobacteria, PG are the only phospholipids [44]. The thylakoid membrane contains ~80% of uncharged glycolipids (~50% MGDG and ~30% DGDG) and ~20% of negatively charged lipids (~10% each SQDG and PG) [11,23,46,50].

Each of these lipids, MGDG and DGDG, has two acyl groups esterified at positions *sn*-1 and *sn*-2 of the glycerol fragment, and a polar head group at position *sn*-3. MGDG contains 1 β-galactose (galactopyranosyl) as a head group. DGDG has a digalactose head group, with the second galactose linked to the first galactose of MGDG by an α1-6 glycosidic bond. SQDG contains 6-deoxy-6-sulfo-α1-glucose as a head group, and PG contains *sn*-glycerol-1-phosphate (Figure 1). Almost every class of thylakoid membrane lipid contains their characteristic FAs in bound form—α-linolenic (18:3^Δ9,12,15^) and cis-hexadecatrienoic (16:3^Δ7,10,13^) in MGDG and DGDG, 16:0 in SQDG and trans-3-hexadecenoic (16:1^3^) in PG [11,13,51].

Thylakoid membranes are composed primarily of proteins, up to 75% of their area [52], with only small areas of lipid bilayer. This limits the efficiency of electron transport in the bilayer, which depends on the diffusion of low molecular weight mobile carriers of electrons. Quinones are among the electron carriers, participating in electron transfer between large protein complexes, while quinone-plastoquinone pairs also couple electron fluxes with movement of protons across membranes. The structure of membrane with embedded proteins should allow the fast diffusion of quinones across the membrane from their reduction sites on one side of the membrane and their oxidation sites on the opposite side [25,36]. Apart from photosynthetic super complexes and related to photosynthesis protein complexes, several groups of ion channels and transporters are localized in thylakoid membranes where their activities have been recorded [53,54,55].

## 3. Photosystems and Antenna Complexes for Light Harvesting

### 3.1. PSII and LHCII

During oxygenic photosynthesis, PSII and PSI operate in a sequence and are tightly coupled to ensure efficient electron transfer under illumination [52,56,57,58]. Both photosystems are chlorophyll-binding protein complexes that also include carotenoids, pheophytin, plastoquinone, lipids, and other cofactors [4,59]. From a functional point of view, PSII is a plastoquinone photooxidoreductase [56], which catalyzes the light-induced reduction in plastoquinone to plastoquinol and oxidation of water to molecular oxygen and protons. To study the structure of PSII, so called BBY membranes are used as a unit of thylakoid membrane, which are enriched in PSII and represent highly purified membrane fractions of grana with a diameter of 300–500 nm [48]. Advances in cryo-EM have allowed high-resolution structures of PSII complexes from various photosynthetic organisms to be obtained [24,42,50,56,58,59,60,61].

In native thylakoid membrane, most PSII RCs exist as dimers. However, monomeric PSII complexes can also be observed. The central protein subunits are the D1 (PsbA) and D2 (PsbD) proteins, which form a heterodimer that binds most of the cofactors involved in electron transfer in PSII [62]. According to a structural model of a typical cyanobacterial PSII, each monomer contains 16 internal and three external transmembrane (TM) protein subunits, 35 Chl *a* molecules, 2 pheophytins, 11 β-carotenes, 2 heme molecules, 1 non-heme iron molecule, 4 calcium ions, 3 chloride ions, 2 plastoquinones, more than 20 lipid molecules, and a metal cluster Mn_4_Ca that oxidizes water to molecular oxygen [58,60]. The total molecular weight of the cyanobacterial PSII monomer is 350 kDa [60].

The core of cyanobacterial PSII of *Thermosynechococcus elongatus* is formed by the two protein subunits D1 and D2; each of them is folded into five TM-α-helices, which are arranged in a semicircular structure and connected by short loops on the stromal side and elongated loops on the luminal side. In D1/D2 heterodimer, each of the five TM helices is linked to one another and ligates cofactors required for charge separation. From lumen to cytoplasm/stroma, two pairs of Chl *a* molecules, one pair of pheophytin molecules, two plastoquinone (PQ) molecules (Q_A_, Q_B_), and nonheme iron (Fe^2+^) are located on the pseudoaxis linking the two subunits extending across the membrane. In addition, two peripheral Chl as well as two β-carotene molecules are associated with the D1/D2 heterodimer [61].

More than 40 proteins are known to stably or transiently associate with PSII [42]. Each plant PSII monomer contains a core complex consisting of four large internal subunits (D1, D2, CP43, and CP47), twelve low-molecular-weight membrane-spanning subunits (PsbE/F/H-M/Tc/W/X/Z), and four external subunits anchored at the luminal surface (PsbO/P/Q/Tn) (Figure 2 of [24]). The major internal subunits (D1, D2, CP43, and CP47) of spinach PSII core are highly similar to cyanobacterial PSII subunits [24]. Indirect interactions involving PsbM or PsbT subunits and PsbO/U/V proteins stabilize PSII assembly and activity in *Synechocystis* sp. PCC 6803 [58].

In most eukaryotes, from various algae to higher plants, TM proteins belonging to the family of light-harvesting Chl *a*/*b*-binding proteins form LHCII and LHCI, which are linked to the core complexes PSII and PSI, respectively, and are encoded by *Inc* gene superfamily. PSII-LHCII and PSI-LHCI supercomplexes are formed in the way [11,15,20]. LHCII plays a key role in photosynthesis by harvesting sunlight and efficiently transferring excitation energy to RCs. LHCII of green plant chloroplasts is the most abundant membrane protein on Earth [46]. At the same time, LHCII serves to protect the photosynthetic apparatus from damage by excessive light and to organize the granular structures of thylakoid membrane [23,46]. While the core complexes PSI and PSII are highly conserved, the systems of external antennae vary markedly between different organisms [63]. LHCII monomers assemble into trimeric complexes, which increases their thermal stability. Plant PSII, together with the peripheral LHCII antennae, form PSII-LHCII supercomplexes, which consist of the major LHCII trimers and minor monomeric LHCIIs, which are CP29, CP26, and CP24 (detailed original pictures of the structures are given in [4,24,64,65]). These supercomplexes are divided into two types of major trimers, C_2_S_2_ and C_2_S_2_M_2_ (C is the core; S and M are strongly and moderately bound LHCII) [64,66]. The two types of major LHCII trimers are linked to PSII core via CP29/26/24. Each monomer of PSII-LHCII supercomplexes contains numerous lipid molecules in addition to protein and pigment molecules [23,46].

The antenna system of PSII in higher plants consists mainly of six Lhcb proteins (Lhcb1-6), encoded by *Lhcb1-6* genes. Lhcb1-3 are organized as homo- or heterotrimers to form the core LHCIIs. On the other hand, the core trimer of LHCII is encoded by nine *Lhcbm1-9* genes [64]. The 25 kDa LHCII apoprotein contains three TM helices and two amphiphilic helices that non-covalently bind eight Chl *a*, six Chl *b*, four carotenoids and two lipid molecules in a tightly packed manner (resolution 2.5 Å). In green algal PSII-LHCII complex, in addition to the core LHCII trimers, only two minor antennae, CP26 and CP29, were found [64].

The structure of PSII-LHCII supercomplex from spinach was resolved using cryo-EM (resolution 3.2 Å). Two similar monomers made the core of the complex; each of the monomers consisted of 25 protein subunits with 105 molecules of chlorophylls, 28 carotenoids and other cofactors. Among the protein subunits, three (PsbO/P/Q) of four at the luminal side formed a triangular crown-like structure protecting Mn_4_CaO_5_-binding domains of CP43 and D1; this is presumably required for optimal oxygen releasing function of PSII. Each monomer of PSII core was connected to one trimeric and two minor monomeric LHCIIs via interactions with protein subunits PsbW/H/Z and interfacial lipid molecules [24].

### 3.2. Photosystem I and LHCI

PSI is a protein supercomplex that catalyzes light-dependent oxidation of plastocyanin (cytochrome c6) and the reduction in ferredoxin. This catalytic reaction is mediated by the TM electron transport chain, which consists of a primary electron donor (a special pair of Chl molecules) and electron acceptors and three Fe_4_S_4_ clusters [67].

The oligomeric state and subunit composition of PSI vary among photosynthetic organisms. Higher plants possess monomeric PSI, while monomeric, trimeric, and tetrameric oligomers occur in cyanobacteria [12,27]. Trimerization may be a way to modulate light harvesting under changing light conditions. The first crystallized and solved by XRC PSI of the thermophilic cyanobacterium *Synechococcus elongatus* exists as a trimer. One monomer contains nine protein subunits with TM-α-helices (PsaA/B/F/I/J/K/L/M/X) and three stromal subunits PsaC/D/E (detailed original figures of the structures are in [14]). The large PsaA/B subunits are linked by a pseudo-axis C_2_ located in the center of PSI monomer and oriented parallel to C_3_ axis. The other subunits are located at the periphery of PSI core. Each PsaA/B contains 11 TM-helices, which are divided into an amino acid domain (6 α-helices) and a carboxy-terminal domain (5 α-helices) [14].

Thus, the luminal surface of PSI is mainly formed by loop regions connecting the TM-helices of PsaA/B subunits. In addition to lumenal loops of PsaA/B, the PsaF subunit with two hydrophilic α-helices provides structural features to this surface of PSI. The loops on stromal surface of PsaA/B partially form a binding interface with PsaC/D/E subunits. These subunits are arranged closely together in a crescent shape, with PsaD closest to C_3_ axis, PsaC in the middle, and PsaE further from the axis. They surround a groove on PsaA that is predicted to serve as a ferredoxin-binding site [14]. The PsaL subunit seems to be vital for the formation of the larger oligomeric complexes since the addition of a single histidine amino acid to C-terminal end of PsaL disrupted trimerization in *Synechocystis* sp. The first obtained crystal structures of PSI from *T. elongatus* and *Synechocystis* sp. demonstrated that the C-terminus of PsaL coordinates Ca^2+^ ion together with PsaL subunit of an adjacent monomer. This probably stabilizes trimer formation and emphasizes the importance of PsaL C-terminus in oligomerization [20,25,49].

Each monomer of the trimeric PSI complex of *T. elongatus* consists of a central heterodimeric electron transfer domain, a photochemical charge separation site, and peripheral light-absorbing domains that contain the bulk of the cofactors—Chl and carotenoids. In PSI, the heterodimer consists of C-terminal regions of two polypeptides known as PsaA/B. Each of N-terminal regions of these polypeptides has six TM helices and binds a large number of antennal Chl and carotenoids [25,68]. The core PSI of the green alga *Dunaliella salina* consists of only seven subunits (PsaA–F and PsaJ) and forms the smallest known PSI. The PSI core of plants and the alga *Clamidomonas reinhardtii* is larger and consists of 14–16 subunits [20,25,69].

The composition and number of LHCI subunits surrounding the PSI core vary significantly among organisms [4,70]. The structure of PSI–LHCI from two species of green algae, microalga *C. reinhardtii* and macroalga *Bryopsis corticulans*, involves an association of up to ten Lhca subunits around the PSI core [2,71]. PSI–LHCI supercomplex of higher plants consists of 16 subunits that coordinate 192 pigments (detailed structures are given in [49,71]). This supercomplex contains four LHCI protein subunits (Lhca1-4) arranged in a semicircle around PSI core [2,33]. Other Chl *a*/*b*-binding proteins, Lhcb4-6, also called CP29, CP26, and CP24, exist as monomeric antennas. The PSI core interacts primarily with the semicircular belt of LHCI via three small PsaG/F/K subunits; there is a wide gap between PSI core and LHCI subunits [49,70]. PsaG interacts stronger with Lhca1 via their helices than the interactions between PSI core and the other LHCI subunits. The PsaF subunit plays an important role in binding Lhca4; without it, it is difficult to form a complete PSI-LHCI supercomplex. Sixteen protein subunits with 10 to 27 lipid molecules have been identified in the plant PSI-LHCI structures ([20] and references therein).

PSI and PSII balance the distribution of light energy absorbed by their LHCs by state transitions to maintain maximum photosynthetic efficiency and prevent photodamage. In state 2, part of the LHCII moves to PSI, forming the PSI-LHCI-LHCII supercomplex. In the green alga *C. reinhardtii*, this supercomplex binds two LHCII trimers in addition to 10 LHCI subunits [63].

## 4. Participation of Polar Lipids in the Functioning of Photosynthetic Apparatus

### 4.1. The Need for Glycerolipids for Photosystems

It has been found that all lipids directly bind to complexes such as PSII, PSI and Cyt b_6_f and, therefore, may be required to maintain their integral functions [29]. The need for galactolipids for oxygenic photosynthesis is obvious; MGDG and DGDG rule in the plant world [6,72]. It is necessary to understand to what extent the thylakoid leaders—MGDG and DGDG are indispensable and why? It is known that the pathways of galactolipid biosynthesis are definitely different in plants and cyanobacteria. Thus, some species of cyanobacteria accumulate large amounts of monoglucosyldiacylglycerols (MGlcDAG) under standard growth conditions [73,74]. However, for some reason, this lipid did not find applications in plants; unlike cyanobacteria, MGlcDAG is not synthesized in plants (Figure 1B). According to other data, mutants of *Synechocystis* sp., where the gene encoding epimerase converting MGlcDAG into MGDG is disrupted, accumulated this glucolipid instead of MGDG and DGDG. Its presence was sufficient to develop the structure of thylakoid membrane and for the realization of photosynthetic activity without galactolipids [72]. In mutants of *Arabidopsis thaliana* with a deficiency of DGDG, the thylakoid membrane of leaf chloroplasts was strongly curved, with large areas in the stroma free of thylakoids. The distorted thylakoid structure in mutants could be restored by transgenic expression of bacterial glucosyltransferase and subsequent accumulation of glucosylgalactosyldiacylglycerols (GlyGalDG), which are similar in structure to DGDG [6]. However, biosynthesis of GlyGalDG instead of DGDG in *A. thaliana* failed to fully restore the functionality of PSII [75]. Consequently, the glucose ring in GlyGalDG is inferior to the structurally similar galactose ring of DGDG and is not able to replace it. This probably occurs simply because of the difference in the location of one of –OH groups outside the ring (Figure 1D,E).

The galactose and glucose rings can be in α- and β-configurations, and neither has a clear advantage in lipids. In MGDG, the β-galactose ring is involved, in DGDG, the β-galactose ring is also located next to glycerol, but for some reason, the α-galactose ring is located in the position farthest from glycerol [51] (Figure 1A,D). In galactosyl glucosyl-DG [36], as well as in DGDG, the position farthest from glycerol is occupied by α-galactose, while the glucose ring is closer to glycerol and, moreover, in α-configuration. These features can be of considerable importance for the contacts of the indicated lipids with protein and other molecules in photosynthetic complexes.

It is known that during phosphorus starvation, plants synthesize other anionic glycolipids—glucuronosyl diacylglycerols (GlycurDAG), although it has not yet been clarified whether they can partially replace PG, like SQDG, under conditions of phosphorus deficiency [52,76]. In addition to PG, another phospholipid, PI, was found as a minor component in plant thylakoid membranes [77]. Its molecule, like PG, has one negative charge, and the myoinositol alcohol ring is similar to galactose ring (Figure 1C). However, the six-membered galactose ring probably has some advantage over the 6-membered myoinositol ring [1,23,76]. Thylakoid membranes can sometimes also contain traces of PC, which, unlike PG, has one positive charge [74]. Still, the reports of PI and PC in plant thylakoid membranes are scarce and require further studies with isolated and purified preparations of thylakoids.

Why do the other lipids (apart from MGDG, DGDG, SQDG, and PG) not participate fully in photosynthesis? Why are trigalactosyldiacylglycerols, which have a larger head group than DGDG, not used [78]? Surprisingly, thylakoid membranes do not require the presence of sterols and sphingolipids, without which the existence of lipid rafts, which are an integral part of the plasma membrane and a number of other membranes of plant cells, is impossible [9].

Comparison of the molecular structure of four selected types of lipids involved in photosynthesis with their potential substituents (Figure 1) shows that the galactopyranosyl ring of MGDG is not different from the same ring of cerebrosides, and two 6-membered rings of DGDG have the same structure as rings of ceramide dihexoside or two rings of β-sitosterol [51]. PG molecule can be represented as consisting of five parts: OH-CH_2_-, -CHOH-CH_2_-, -O-PO_2_-O-, glycerol, and FA chains. In addition to PG, glycerol is a part of a number of glycerolipids, and the FA residues of both PG and other lipids involved in photosynthesis require special consideration. A phosphate group is characteristic of all phospholipids, and it is difficult to say yet what is special about the -CHOH-CH_2_- group. Finally, the outer part of the head group (OH-CH_2_-) of PG is also present in cholesterol (dominant in animal membranes) or in alkyl esters of glycerol, both of which, however, have no charge [51]. Probably the most important difference between PG and other phospholipids is the absence of a nitrogen atom with its positive charge in its head group (Figure 1G). The presence of exactly one, and precisely a negative charge in PG molecule turned out to be important for the protein components of photosystems, and what the secret is here is yet to be discovered. Why photosystem proteins “don’t like” lipid molecules that are positively charged or have two different charges is still unknown.

In those rare cases when thylakoids contain PI, it would be good to understand functions of which of four selected lipids it performs there (Figure 1C). PI has a number of properties inherent in this quartet: in terms of the 16:0 content, it is the same as SQDG; in terms of the absence of nitrogen and negative charge, it is the same as PG; and in terms of the structure of head group ring, it is close to MGDG. Why is it not good enough for the main roles in photosystems?

The competition of lipid species for participation in photosynthesis presumably took place in cyanobacteria 2.5 billion years ago when the four known lipid species that are still functioning now were selected. It is interesting that numerous lipid species that later became present in eukaryotic cells, in particular phospholipids, did not participate in this competition. These lipid species are still absent from thylakoids of green algae and vascular plants. Thus, DPGs, which surpass MGDG in the ability to form a conical shape of the molecule or PG in the number of negative charges in them, are not in demand in photosystems. The question arises, are they all worse than those four species in geometry of their molecules and their chemical properties, or did they not fit the ancient gene systems of cyanobacteria, which passed on to photosynthesizing eukaryotes? Several explanations are conceivable for selecting the four present lipids: high availability of photosynthetic products such as hexoses in cyanobacteria; lack of excessive phosphate for the lipids; specific biochemical and gene networks and their restrictions required for photosynthesis and others. Interestingly, lipids of heterotrophic prokaryots *E. coli* and *B. subtilis* mostly have polar heads of phosphatidylethanolamine and PG, e.g., [79] while more reasons for choice of lipid species are leading to the origin of life [80]. There is also an opinion that most enzymes involved in biosynthesis of lipids in plant chloroplasts did not originate from cyanobacteria. It has been shown that acquisition of these enzymes by plants, although it occurred from several bacteria, occurred earlier than the transfer of cyanobacterial ribosomes to chloroplasts and, accordingly, genes for photosynthesis [5,10,81].

The participation of MGDG in the construction and functions of thylakoid membranes was discussed above. DGDG is also necessary for the correct formation and maintenance of thylakoid membrane. According to in vitro analysis, DGDG affects membrane adhesion through hydrogen bonds between the polar head groups of these molecules from adjacent bilayers [82]. In this case, the head groups of DGDG containing two galactose rings play a crucial role in thylakoid architecture (Figure 1D) [6]. Although the alkyl chains of DGDG are highly unsaturated (mostly 18:3), DGDG maintains a cylindrical shape due to steric compensation by its bulky head group and forms a lamellar phase (like SQDG and PG) [23].

The total amount of anionic lipids SQDG and PG is maintained constant in both plants and cyanobacteria, which is likely necessary for the proper functioning of the entire photosynthetic apparatus [6,23,83,84]. However, contrary to PG the requirement for SQDG for photosynthesis and growth varies depending on the species [23,78]. In *A. thaliana* and two cyanobacterial species, inactivation of SQDG synthesis genes resulted in only a very minor effect on their growth and oxygen evolution activity [29,85]. *A. thaliana* mutants completely lacking SQDG exhibited wild-type-like phenotypes under nutrient-sufficient conditions [23,85]. Thus, SQDG are not essential for the growth of this plant under optimal conditions [23,52]. At the same time, it has been shown that in *A. thaliana* and cyanobacteria, a decrease in PG content under phosphorus deficiency is compensated by an increase in SQDG [6,23,78,86]. However, it remains unclear what part of the PG functions can be replaced by SQDG under conditions of phosphorus deficiency [86]. Conversely, loss of SQDG in *A. thaliana*, green alga *C. renhardtii*, and several cyanobacterial species due to genetic alterations results in increased PG levels, likely to preserve total anionic lipids [22,78,83,85]. Thus, in *T. elongatus*, due to altered SQDG biosynthesis, the PG content was increased from 4 to 25 mol.% [22,85].

Simultaneous partial reduction in PG content and SQDG deficiency greatly impair photosynthetic activity and growth of most photosynthetic organisms [6,22,78,85,86]. Mutants that completely lack PG and SQDG biosynthesis in plastids showed defects in development of roots, hypocotyls, and embryos in addition to leaves [78]. These data confirm the complementary relationship between SQDG and PG and suggest the importance of maintaining the total amount of anionic membrane lipids for optimal functioning of the photosynthetic apparatus [78,83]. Unlike PGs, which are required for oxygenic photosynthesis in cyanobacteria, algae, and plants, it remains unclear why the importance of SQDG presence varies greatly among individual photosynthetic organisms [85].

Apparently, PG is the only lipid absolutely essential for oxygenic photosynthesis [6,23,39,85,87]. A recent study [88] is devoted to PG biosynthesis. PG plays an important role not only in photosynthesis in general, but also specifically in the activity of PSII and PSI [29]. Growth of mutant *Synechocystis* sp. cells deficient in their ability to synthesize PG in a PG-free medium resulted in a 90% decrease in its content and a 50% loss of photosynthetic oxygen-evolving activity [84]. PG deficiency has a strong effect on thylakoid biogenesis [6,23]. About 30 mol.% of PG in thylakoid membrane is distributed among the photosystem complexes as a structural component in both cyanobacteria and plant chloroplasts, whereas the quantitative contribution of PG to the thylakoid lipid bilayer is small (5–8 mol.%). Therefore, the lipid matrix of membranes is mainly composed of glycolipids [22]. Deletion of PG by inactivation of its synthesis has been shown to inhibit the growth of cyanobacteria, mainly due to a decrease in stability and activity of PSII [29]. Loss of PG severely impairs photosynthetic electron transfer in all oxygen-containing phototrophs studied to date [22,29,89]. Thus, PG may serve as an important component of photosystems, but the functional relationship of PG with SQDG requires further study [83,85]. Since SQDG contained in PSII accounts for only 3.7 mol.% of the total SQDG level in *Thermosynechococcus* thylakoids, PG produced in response to SQDG loss will be mainly distributed in lipid bilayer fraction [22].

Genetic approaches applied to *A. thaliana* have shown the requirement of PG for chloroplast biogenesis. Mutants deficient in PG biosynthesis in chloroplasts had pale yellow-green leaves and failed to develop thylakoid membrane networks within these chloroplasts [86]. Chloroplast PG deficiency in *A. thaliana* mutants results in the formation of vesicles, enlarged vacuolated structures, or underdeveloped membrane fragments. These data confirm that PG is required for photosynthesis in plants [6,86].

### 4.2. Polar Lipids in PS II

The photosynthetic complexes contain numerous glycerolipid molecules, as demonstrated by XRC and cryo-EM. The PSII complexes of plants, green, red and diatoms show similar lipid binding sites in the core region, with the exception of *T. vulcanus*, suggesting a highly conserved role for these lipids.

A variety of glycerolipid molecules have been found in PSII and PSI of various organisms [10]. Accurate determination of binding sites and identity characteristics of these lipids is necessary to elucidate specific interactions between lipid molecules and proteins/cofactors within the complexes [23].

Lipids found in structures of photosynthetic proteins (XRC method) bind light-absorbing cofactors, fill cavities within proteins through which quinones can diffuse, form an important part of monomer–monomer interface in multimeric structures, and can contribute to the structural flexibility of complexes that undergo partial disassembly and reassembly. Individual lipids can influence the biophysical properties of RC cofactors, particularly in the case of quinones, and thus the rate of electron transfer through the complex [25].

It is known that the shape of lipid molecules is determined by the size of head group, the degree of saturation of alkyl chains, and the individual characteristics of these chains. It has been shown that the lipid binding sites in the core of both photosystems are highly conserved among cyanobacteria, algae, and land plants. It is very likely that all lipid molecules in PSII and PSI play independent decisive roles for structures and functions of these photosystems. Pigments and lipids can be integrated into photosynthetic complexes during folding of nascent protein chains and/or assembly of the complexes. It is likely that lipid biosynthesis and photosystem assembly are somehow coordinated, and newly synthesized lipids can be incorporated into photosystem subunits during their assembly. Thus, the specific and extensive distribution of PGs in deep regions of photosystems may require a unique mechanism for incorporation of PGs into complexes, possibly in coordination with the synthesis of photosynthetic proteins and pigments [10,22,46].

Photosynthetic protein complexes interact with lipids through hydrogen bonds and salt bridges. From two to six amino acids interact with each lipid head group (Figure 1). The average number of hydrogen bonds and salt bridges in MGDG and DGDG head groups is 4.5 and 8.5, respectively [61].

All lipids in PSII complexes are oriented with their head groups either toward thylakoid lumen or toward cytoplasm [23,90]. The present data from XCR and cryoEM results show that head groups of most MGDG and all DGDG are located on the lumenal sides with low pH of both photosystems, whereas head groups of SQDG and PG with their negative charges are found mostly on the stromal/cytosolic side of photosystems occupied by the weakly basic cytoplasm [10,13,25,63]. Some MGDG molecules can also be found on the cytoplasmic side [12]. PG head group is located in an environment formed mainly by Phe, Trp, and Arg residues and is able to interact with both D1/D2 domain and CP43 antenna. A close binding of PG to the inner part of D1 membrane was concluded on the basis of lipid analysis of purified D1 protein [23]. It should be noted, however, that ATP-dependent flippase activity for PG changing the position of the lipid between leaflets of the bilayer was found in preparations of thylakoids from leaves of *Spinacia oleracea* [91].

Lipids play a specific role in stability and interaction of pigment-protein complexes involved in photosynthesis, and this is of particular importance during the reassembly of photodamaged PS II complexes [92]. In PSII dimer, 1 MGDG and 2 SQDG molecules are present at the interface of two monomers, which may indicate that they facilitate tight molecular interactions between the monomers [52].

A functionally significant role for MGDG and PG may be that these lipids are of the correct size, shape, and charge to fill empty spaces in protein structure, facilitating tighter contact between adjacent electron transfer and light absorption domains. The need to provide a structurally flexible environment near Q_B_ site requires the presence of some lipids covering the D1/D2 core, but this does not explain the presence of lipids bound elsewhere on D1/D2 surfaces. For example, the tail surrounding Q_A_ plastoquinone during its exits from D1/D2 core is composed of two MGDGs, although this quinone is not released from RC during electron transfer. It is proposed that the function of the lipid coat of D1/D2 core is to provide structural flexibility that allows the replacement of photodamaged D1 polypeptides. In PSI, such a selective repair of the core polypeptide does not occur, and here the lipids are replaced by pigments [52].

The number of lipid molecules of the selected four classes in PSII monomers of six species of phototrophs (cyanobacterium *Thermosynechococcus vulkanus*, two species of algae, and three species of land plants) was given earlier [20] and is in the range of 14–25 molecules, with MGDG and PG predominating. The variability is mainly explained by the variability between biological species. The number of lipid molecules in other photosynthetic complexes of photoautotrophs is given in Table 1. Below we present the number of lipid molecules in the series MGDG, DGDG, SQDG, PG in the complexes as a digital ratio, according to the given sequence, for example, 1:1:1:1.

Analysis of the crystal structure of the PSII dimer from *T. vulcanus* at 1.95 Å revealed 20 lipid molecules per monomer (6:5:4:5) [15,20]. The head groups of lipids in PSII are asymmetrically distributed throughout the complex according to the tendency described above (nonpolar MGDG and DGDG are to the lumenal side while polar SQDG and PG heads are to the stromal side), and only one MGDG molecule faces head group to cytoplasmic side. Of the lipids of monomer, 13 (3:4:1:5) are in close proximity to D1/D2 heterodimer, while 3 (1:0:2:0) are located at monomer interface and 4 (2:1:1:0) are at the periphery of PSII [10,20,23]. Near RC, three PG and one PG are located near the primary (Q_A_) and secondary (Q_B_) quinone binding sites, respectively, while seven molecules (2:3:1:1) are located at the interface between D1 and CP43. Based on the localization of the lipids, they are likely to form a hydrophobic pocket for plastoquinol-plastoquinone exchange and promote the interactions between protein subunits. A large number of lipid molecules around PSII provide structural flexibility of RC and participate in the turnover of protein subunits, especially D1 during repair of damaged PSII. PSII is thought to assemble as a dimer but is able to function in a monomeric form in vivo [93]. Analysis of the *T. vulcanus* PSII monomer in another study revealed only 13 lipid molecules (3:4:2:4) in the complex [1].

The structure of dimeric PSII of the cyanobacterium *T. elongatus* (XRC method, 3.0 Å resolution) was previously determined to include 14 lipids per monomer (6:4:3:1) [83,90]. Of these lipids, 11 are located at the periphery of D1/D2 core heterodimer and appear to form an interface between D1/D2 and surrounding LHCII proteins and cofactors within a single monomer [21,25]. These lipids play a role in stabilizing the interaction of LHCII proteins with PSII core [61,90]. Of these, six (2:4:0:0) are located with their lipid head groups on the lumenal side of membrane, and five (3:0:1:1) are located with head group to the stromal side [52].

Later, in the structure of the homodimeric PSII complex (resolution 2.9 Å) from *T. elongatus*, consisting of 20 protein subunits, the presence of not 14, but 25 lipid molecules per monomer was discovered (Table 1), which makes PSII the most lipid-rich membrane protein complex. These lipid molecules serve as multifunctional cofactors involved in the assembly and functional regulation of PSII [13]. Head group of one of SQDGs is located in a positively charged pocket between D2 and CP47 subunits. On cytoplasmic side, one MGDG is located next to SQDG, at the interface between monomers, and another is located next to PQ-PQH_2_ exchange cavity. Another MGDG is located next to DGDG in a pocket formed by CP43 and the small subunits PsbJ and PsbK [61,90].

**Table 1:** Number of lipid molecules in the major photosynthetic complexes of photoautotrophes.

№	Object	Numbers of all Lipid Molecules in a Monomer and Number for Each of MGDG, DGDG, SQDG, and PG	References
1.	PSII of *Thermosynechococcus elongatus*	A total of 25 molecules in a monomer; 11:7:5:2	[61,90]
2.	PSII of *Synechocystis* sp. PCC 6803	Over 20 molecules	[60]
3.	PSII of *Pisum sativum.*	A total of 25 molecules; 7:5:4:9	based on [20]
4.	C_2_S_2_ PSII of *Chlamidomonas reinchardtii*	A total of 21 molecules; 5:5:2:9	based on [20]
5.	LHCII of *Spinacia oleracea*	Four molecules in trimer; 0:3:0:1. DGDG at the border between monomers	[23,94]
6.	PSII-LHCII of *Spinacia oleracea*	Calculated, 30 molecules per a monomer of PSII; 5:4:3:18	[22]
7.	PSI of *Pisum sativum*	All four lipids and phosphatidylcholine (PC) detected by high performance liquid chromatography/electrospray ionization mass spectrometry	[95]
8.	Cyt b_6_f of *Spinacia oleracea*	A total of 12 molecules in dimer (2:0:3:4 and 3 PC, added exogenously)	[96]
9.	NDH-1 *Thermosynechococcus elongatus*	A total of 13 molecules (2:2:3:6) or A total of 15 molecules (0:2:4:9)	[19,97]

The free space between monomers in *T. elongatus* PSII is filled by 14 lipid molecules, 7 from each monomer. Six of these forms contact the protein subunits of both monomers, and the other six lipids mediate interactions between protein subunit of one monomer and the lipid of the other monomer. Of the 18 extra lipids per monomer which are not involved at monomer–monomer interface, 3 are located at the periphery of PSII, and 7 form clusters with 2–3 lipids in a lipid band around D1 and D2; 2 DGDG between D1 and CP43, a group of 3 MGDG, and a group of SQDG and DGDG between D2 and CP47 [61].

#### 4.2.1. Neutral Glycolipids in PSII

There is no doubt that MGDGs are required for PSII function, since the lipids involved in dimerization of PSII core (*T. elongatus*) are largely composed of MGDG. These include 8 of the 14 lipids located at monomer interface [56]. In an *A. thaliana* mutant with a significant loss of MGDGs, PSII activity was sharply reduced, leading to the death of seedlings. It has been shown that MGDG strongly stimulates dimerization of monomeric PSII, since 2 MGDG are located at the monomer interface of PSII dimer [50].

MGDGs strongly induce dimerization of monomeric PSII from *Spinacia oleacea* in vitro. Therefore, MGDG molecules located at monomer–monomer interface of PSII dimers, which are conserved in cyanobacteria and photosynthetic eukaryotes, can mediate PSII dimerization, and their degradation induces its monomerization during the repair of damaged PSIIs [10,25]. One of the MGDGs found at the dimer interface has its headgroup within 7 Å of Q_A_ quinone headgroup, and they are separated only by an amino acid loop that forms part of the Q_A_ site [21,25].

Lipid–protein interactions keep a vital function in mediating the structural and functional interactions of multisubunits in PSII supercomplexes [52]. MGDG has an important role in maintaining the structural organization of chloroplast membrane and activity of the embedded protein supercomplexes. It is an important factor for PSII activity, as high-resolution structural analysis of cyanobacterial PSII revealed six tightly bound MGDGs around PSII core [21]. MGDGs also play a critical role in violaxanthin de-epoxidation in xanthophyll cycle under high light conditions [52,98].

DGDG is in need for the functional and structural integrity of oxygen evolving complex and the thermal stability of plant PSII. DGDG deficiency reduces the thermal stability of LHCII–PSII containing macrodomains. Moreover, DGDG is also involved in stabilization of plant LHCII trimers; it mediates interactions between adjacent LHCII trimers on the luminal side. In cyanobacteria, DGDG may be involved in the binding of extrinsic proteins to PSII and the stabilization of oxygen evolving complex [23].

Studies of *A. thaliana* mutants lacking DGDG indicate that a subset of DGDG molecules bind specifically to and predominantly affect the reactivity of PSII at the electron donor side. Hydrogen bonding interactions of DGDG with various Tyr residues have been observed in DGDG-depleted PSII [13,21,94].

Disruption of DGDG biosynthesis resulted in dissociation of PSII extrinsic proteins, namely PsbU/V/O, and destabilization of the complex [45]. DGDG molecules are integrated into the luminal part of PSII near the extrinsic proteins and may participate in the assembly of extrinsic proteins and stabilize the complex. Loss of DGDG in *Synechocystis* PCC 6803 caused photoinhibition at high temperature and bright light, in part due to impaired PSII reconstitution [13]. The lipid layer surrounding the PSII core and containing DGDG may serve as a molecular lubricant for the replacement of damaged D1 by newly synthesized protein [45]. The future studies are needed to determine the binding constants and other physico-chemical parameters for interactions of MGDG and DGDG with specific subunits of PSII.

Interestingly, it was recently shown that wild-type and PsbS-knockout *Arabidopsis thaliana* plants behaved differently under illumination: wild-type plants exhibited reversible shrinking of thylakoid membrane from around 4.3 to 3.2 nm, the effect was absent in mutants [37]. Moreover, DGDG was repelled from the LHCII-PsbS complex during the process because of an increase in the p*K*_a_ of lumenal residues and in the dipole moment of LHCII [37].

#### 4.2.2. Anionic Lipids in PSII

SQDG deficiency has different effects on photosynthetic activity in different organisms. It has been shown that SQDG plays a critical role for PSII activity in *Synechocystis* sp. and *C. reinhardtii*, but this is not the case for *T. elongatus* and *A. thaliana* under nutrient-sufficient conditions [29,85]. In a SQDG-deficient mutant of cyanobacterium *T. elongatus*, deficiency of this lipid partially attenuates PSII activity by disrupting secondary quinone exchange (Q_B_) at acceptor site. Replacing two SQDGs located at the monomer–monomer interface by other lipids reduced the stability of the PSII dimer, resulting in an increase in amount of PSII monomer in the mutant. SQDG binding at all sites in PSII is presumably required to maintain the activity and stability of PSII; replacement of SQDG by other lipids can only partially compensate for its functions [29,94].

Each PSII monomer binds four SQDG molecules. It was shown that SQDG was present on the outer surface of tobacco D1/D2 heterodimer, whereas PG was tightly associated with the inner part of D1. High-resolution structural analysis of PSII dimer of thermophilic cyanobacterium *T. vulcanus* also revealed the binding of four SQDG molecules in PSII monomer. Among them, two are located at the monomer–monomer interface, the third at the interface between CP47 and D2 proteins and near the quinone electron acceptor Q_B_, and the fourth in the region between PsbX and PsbF (β-subunit of Cyt b559). This cytochrome is involved in the secondary electron transfer pathway, protecting PSII from photoinhibition. In the structure of *T. elongatus* PSII monomer, one of these two SQDGs located at the monomer–monomer interface of the dimer was not detected, but this SQDG may be required for dimerization of PSII monomer during assembly [29].

Mutants of *Chlamydomonas* and *Synechocystis* sp., unable to synthesize SQDG, have usual amounts of PSII but reduced PSII activity. In preparations of PSII from this *Chlamydomonas* mutants, their activity can be restored by addition of exogenous SQDG, and the loss of PSII activity is apparently due to deficiencies in electron transfer from water to TyrZ on the donor side of PSII [52,70,99,100]. Three SQDGs found in cyanobacterial PSII structure (XRC method) are located on the opposite side of membrane compared to Mn_4_Ca cluster and TyrZ. One of them is located close to PG and is attached to the surface of D1, while the remaining two are located on the surface of CP47 at dimer interface [52].

The SQDG and PG binding sites at the monomer–monomer interface of PSII dimer are also conserved in cyanobacteria, and genetic loss of biosynthesis of these lipids also leads to a disruption of dimerization in the latter. Thus, these lipids are necessary for monomer–monomer interaction of PSII in both plants and cyanobacteria [10].

An important structural and functional role of PG is the formation of PSII dimers from monomers by their incubation with PG, as well as the stabilization of these dimers [99]. In dimeric PSIIs of spinach, PG was found to be located at the dimerization interface. It has been shown that treatment of spinach PSII dimers, pea LHCII trimers, and *A. thaliana* PSII-LHCII complexes with phospholipase A2 resulted in PG cleavage in all of these complexes and their disintegration into monomeric forms [1,20,94,101,102]. Upon addition of PG, monomerized spinach PSII was converted back into dimers, and the reduced level of trimeric LHCII in a PG-deficient mutant of *C. reinhardtii* was restored [20]. PG molecules stabilize PSII dimers in *Synechocystis* sp. and facilitate the binding of inner antenna protein CP43 to PSII core. According to crystal structure of PSII, two PG molecules are located at the interface between CP43 and the D1/D2 heterodimer [39,90]. The PG head groups are surrounded mainly by phenylalanine, tryptophan, and arginine residues and are able to interact with both D1/D2 domain and CP43 protein [20,51]. In both cyanobacteria and plants, PG deficiency not only impairs the assembly and stability of PSII complexes, but also the electron transport between the primary and secondary quinone acceptors within the PSII RC. PG has been shown to be necessary for electron transfer on both the donor and acceptor sides of PSII in cyanobacteria and plants [20]. Characterization of PG-deficient mutants in *Synechocystis* revealed that PG is required for various photosynthetic processes, such as electron transfer from primary plastoquinone (Q_A_) to secondary plastoquinone (Q_B_) at PSII acceptor side [6,10,93], PSII reactivation after photoinhibition, and maintenance of oxygen evolution complex activity at the PSII donor side [6,103,104].

Analysis of mutants in *Chlamydomonas* demonstrated that PG is required for synthesis of the major PSII proteins (D1 and CP47), the formation of LHCII trimers, the transition to another state, and the oxygen evolution activity. In a PG-deficient *Arabidopsis* mutant, photochemical efficiency of PSII was significantly reduced, and the further loss of PG due to phosphorus deficiency resulted in the absence of PSII activity [6,10].

In the crystal structure of PSII dimer containing 20 lipid molecules per monomer [60], 5 are PGs. In this structure, three PG molecules surround Q_A_-binding site at the monomer–monomer interface. The other two PG molecules are located near Q_B_-binding site in the region between CP43 and D1/D2 heterodimer, respectively. One of the latter PGs interacts with α-subunit of PsbE (Cyt b559), which forms a heme-bridged heterodimer with β-subunit of PsbF and functions as an essential component of PSII [60,105].

It has been shown that PG is required for the function of plastoquinone electron acceptor Q_B_ in PSII reaction center of oxygenic photosynthetic enzymes [84]. PG is involved in the association of external proteins and the maintenance of structure and function of the Q_B_-binding site in PSII. When PG molecules located near the Q_B_-binding site were lost in a mutant, the function of Q_B_ was impaired [10,62]. Lipid analysis also showed that six PG molecules are present in wild-type PSII, whereas approximately half of the PG molecules are lost in PSII of PG-depleted mutant, which negatively affects the donor side of PSII [13,62,103]. In PSII mutant complex, the outer proteins PsbO/V/U, required for stabilization of Mn–Ca cluster, were dissociated due to the lack of PG [62].

The known structures of several photosynthetic complexes from cyanobacteria and plants led to estimates that about 6–8 mol % of the total thylakoid lipids are linked to these complexes. Interestingly, PG molecules in thylakoids demonstrate preferential localization in photosystems with at least ~30% of the total PG molecules in them. This coincides with the special importance of PG for photosynthetic electron transport in cyanobacteria and plants [10,22,86].

In addition to a possible role in monomer–monomer interactions, one of the PG molecules plays a direct role in cofactor binding. The environment of this lipid actually consists of a loop of the PsaA polypeptide, a carotenoid, and macrocycles of three Chl molecules. This PG interacts directly with one of these Chl [52].

### 4.3. Polar Lipids in LHCII and PSII-LHCII

In addition to protein and pigment molecules, each monomer of PSII-LHCII supercomplex also contains numerous lipid molecules [23,24].

The structure of the plant PSII–LHCII supercomplex (high-resolution cryo-EM) type C_2_S_2_M_2_ of *Pisum sativum* revealed 34 lipid molecules per monomer of homodimeric system [64]. MGDG, DGDG, and SQDG molecules are mainly located in PSII core. MGDG molecules are distributed around CP43, CP47, D1, and D2 subunits, while DGDG molecules are concentrated in the inner region of the core around D1 and D2 subunits. Three SQDG molecules are located at dimerization interface of PSII core, and one SQDG is located in the peripheral cavity surrounded by CP43, PsbK/J, Cytb559 (PsbE/F), and D1. There are nine lipid molecules per monomer (2:1:3:3) at dimerization interface of PSII core. PsbTc/L/M trimer is linked to symmetrical PsbTc’/L’/M’ trimer via extensive hydrophobic interactions between PsbM and PsbM’. These subunits are located at dimerization interface of PSII core dimer and are stabilized by seven lipid molecules (1:0:3:3) [23]. These lipid molecules play a crucial role in stabilizing the oligomerization interfaces of plant PSII dimer and LHCII trimer. Moreover, they also moderate interactions between PSII core subunits and promote assembly between the peripheral antenna complexes and PSII core [23].

Cryo-EM analysis of the C_2_S_2_-type PSII–LHCII supercomplex from *Spinacia oleracea* (spinach) revealed 16 lipid molecules (5:4:3:4) in the PSII core per monomer, as well as 5 PGs in LHCII antenna. Similarly, analysis of pea C_2_S_2_M_2_ type PSII–LHCII supercomplex revealed 25 lipid molecules (7:5:4:9) in PSII core and 9 PGs in LHCII antenna [64]. The lipid binding sites in the core of pea PSII dimer are very similar to those in *T. vulcanus* PSII [23]; 9 lipid molecules (2:1:3:3) are located around the interface of monomer-monomer core and are required for dimerization, whereas six lipid molecules (2:2:1:1) are located near Q_B_ site and form a hydrophobic cavity with bound protein subunits. In addition, two molecules (MGDG and DGDG) located near the fifth TM helix of CP43, which is involved in the assembly of CP43, D1, and PsbI, and one DGDG molecule near the same helix of CP47, which stabilizes the assembly of CP47, D2, and PsbH, are also common to pea and *T. vulcanus* [23]. However, 7 lipids (2:0:0:5) located in the peripheral regions of CP43 and CP47 that are involved in the assembly of CP26/LHCII/CP29 and CP43/CP47 are absent in *T. vulcanus* PSII, which is consistent with the absence of LHCII antennae in the cyanobacteria. Furthermore, the PG binding site observed near Q_B_ site of *T. vulcanus* PSII is not conserved in pea PSII.

Nine PG molecules have been found in LHCII antenna complexes: three in S-LHCII trimer, three in M-LHCII trimer, and one in each of CP29, CP26, and CP24 monomers per PSII monomer [64]. The three PGs in S-LHCII and M-LHCII are located at the interface between the monomers and participate in trimerization. Amino acid sequence WYXXXR in LHCII, which is required for PG binding, is essential for trimer formation, as demonstrated by the dissociation of the trimer into monomers upon removal of N-terminal domain containing this sequence and the loss of PG molecule [106]. PG molecule in CP26 mediates CP26–CP43 interaction at the interface between these two proteins, while PG in CP29 stabilizes the CP29–CP24 heterodimer at the interface between these two proteins. subunits [23]. PG molecule in CP24 is located in the peripheral region and can facilitate the association of two adjacent C_2_S_2_M_2_ complexes into megacomplexes [20].

It has already been mentioned that LHCII is involved in key interactions with lipids, since the trimeric form of LHCII is tightly associated with PG, and removal of PG causes dissociation of LHCII trimers into monomers [25,94]. Each trimer of pea LHCII complex (2.5 Å resolution) includes three DGDG molecules that occupy a cavity at the three-fold symmetry axis on the luminal side of membrane. The structure of the trimeric pea and spinach LHCII complexes (XRC method) includes a PG molecule located at the monomer interface, which provides an axial ligand for Chl. This PG plays a direct structural role in the binding of one of antenna Chl molecules [101].

LHCII, in addition to its primary functions, is responsible for a non-photochemical quenching process that dissipates excess excited state energy under high irradiance conditions. The LHCII monomer consists of a single polypeptide with three TM-α-helices; this provides a scaffold for the binding of 14 Chl and four carotenoids. The three monomers then assemble into a trimeric structure [52,107]. The structure of spinach complex (XRC method) includes three DGDG molecules per trimer, which facilitate contacts with neighboring trimers in the crystal lattice [23,94].

The work [64], devoted to PSII-LHCII supercomplex of C_2_S_2_M_2_ type of *Pisum sativum*, provides data on the formation of ionic and hydrogen bonds, van der Waals and hydrophobic interactions by lipid molecules, in which such amino acids of TM-helices as Arg, Asn, Asp, Ala, Glu, His, Leu, Lys, Thr, Trp, Tyr, Ser are involved. FA chains form hydrophobic interactions not only with non-polar amino acid residues, but also with β-carotene molecules and phytol residues of Chl molecules [64].

### 4.4. Involvement of Polar Lipids in the Assembly and Functioning of PSI

Mass spectrometry of PSI has shown that individual molecules of the known lipid quartet with the addition of PC are associated with PSI complex. PSI catalyzes the transfer of electrons from lumenal plastocyanin to stromal ferredoxin using the energy of absorbed photons. In this process, both interactions between protein molecules and between proteins and lipids play a significant role [95].

The number of lipid molecules from four selected classes in PSI monomers of seven species of cyanobacteria, six species of algae and three species of land plants was given earlier [20]. Cyanobacteria usually include three–six lipid molecules with a predominance of PG (two–three molecules), while DGDG and SQDG are often completely absent. PSI of algae contains 3–13 lipid molecules, also with a predominance of PG (two–seven molecules). The PSI of land plants contains five–six lipid molecules with a predominance of the same PG and the absence of SQDG. The content of lipid molecules in PSI and PSI-LHCI complexes is given in Table S1 of [20].

In the trimer of *T. elongatus* PSI, containing four lipid molecules per monomer, a pair of MGDG and PG molecules are located symmetrically near the core, which indicates the role of these lipids in the formation of center of photochemical reactions. One PG molecule is located at the interface between the monomers, which may be important for trimerization of PSI; another PG is at the site of contact with PsaX [20]. A similar configuration of lipid molecules is observed in many cyanobacteria, indicating their highly conserved function in PSI.

Each monomer of cyanobacterial PSI is associated with 127 cofactors, four of which are represented by lipids, one molecule of MGDG and three molecules of PG [22,52]. The two lipids, MGDG and PG, are symmetrically located at opposite ends of the central electron transfer domain on the stromal side of membrane, filling the space between this domain and the adjacent light collecting sites; the result is that these lipids are located deep within PSI monomer [52].

Analysis of the structure of cyanobacterial PSI shows that the head group of negatively charged PG is located 15 Å from the phylloquinone of A-branch, while the neutral MGDG is located 15 Å from the phylloquinone of B-branch. It is suggested that these features of the lipid environment may contribute to the difference in energetics of quinone for electron transfer in the two branches. The different chemical properties of these molecules may account for the tenfold difference in the rate of electron transfer along these two branches of cofactors [44,52,108].

MGDG is located at the luminal side, filling the space between helix C of Lhca4 and the interface between PsaF and PsaB. The molecule is located between Chl *a*/Lhca1 and Chl *a*/PsaF, which are important for in energy transfer. The other two lipid molecules are MGDG/PsaG and PG/Lhca1. MGDG/PsaG molecule is located lateral to PsaG, and PG/Lhca1 is located between helix C of Lhca1 and PsaG and interacts with Glu-29 residue of PsaG. PG molecule is located close to the N-terminal loop of Lhca4 at the stromal side [108].

The use of MGDG on one side of the complex and PG on the other side may reflect certain differences in the shape and surface charge of the surrounding protein structure. MGDG and PG may also play a structural role in the internal contact surfaces between proteins in PSI monomer. Two additional PGs found in cyanobacterial PSI structure are located at the periphery of each monomer and are symmetrical with respect to the central PsaA/B heterodimer. There is evidence that these PG molecules are involved in PSI biogenesis and stabilization of PSI trimers in cyanobacteria. In particular, partial loss of PG in a strain of *Synechocystis* sp., in which PG synthesis is impaired, was found to result in a decrease in overall PSI activity and a loss of trimeric form of PSI in favor of monomeric form [39,52,62,109].

In PSI complexes from *Archaeplastida* and *T. elongatus*, three PG molecules are located in the core. MGDG molecule near the reaction center in cyanobacterial PSI is replaced by DGDG in *Archaeplastida*. PSI structure of the diatom *Chaetoceros gracilis* and the cryptophyte *Chroomonas placoidea* also has a DGDG molecule symmetrically to PG in PSI core, suggesting that the DGDG binding site in PSI has been strictly conserved during the evolution of photosynthetic eukaryotes. In a DGDG-deficient mutant of *A. thaliana*, the number of core PSI proteins is reduced compared to the wild type [110]. Thus, the DGDG molecule on the stromal side of PSI may function to stabilize the interaction between the stromal subunits of PSI and the core proteins [10,110].

Amino acid residues around the lipid binding sites strongly influence the affinity of lipids for these sites. In both cyanobacteria and plants, PsaB subunit of PSI complex, namely its N- and C-terminal regions, interacts with galactose moiety of MGDG or DGDG. The amino acid residues of PsaB are located near the second galactose moiety of DGDG, so they can influence the selectivity of galactolipids in this region. In plants, C-terminal region of PsaC subunit also partially interacts with the polar head group of DGDG. In PSI, PsaC interacts with the second galactose moiety of DGDG. In PSI from *Synechocystis* or *Pisum sativum*, the amino acid residues of PsaB and PsaC interact with the galactolipid molecule [3].

In the structure of PSI trimers from *T. elongatus* [10] (2.5 Å resolution), only four molecules per monomer (1:0:0:3) were identified along with many protein subunits, pigments, and other cofactors. One molecule of MGDG and one PG molecule were located symmetrically near the PSI core, suggesting an important role of these lipids in the formation of PSI. Of the other two PG molecules, one is located at the monomer–monomer interface, which may be important for PSI trimerization, while the other one is located at the contact site with PsaX. Similar lipid binding sites in PSI are demonstrated by various cyanobacterial species [10]. DGDG has also been shown to play an important role in the functioning and stability of the PSI complex. Structural analysis of the *T. elongatus* PSI core at 1.97 Å resolution revealed DGDG instead of MGDG. Thus, the MGDG binding site in cyanobacterial PSI may be able to bind DGDG instead of MGDG. Lipid analysis of PSI trimer isolated from *Synechocystis* sp. revealed six lipid molecules per monomer (2:1:1:2), suggesting that DGDG is also integrated into PSI structure [10,108].

In cyanobacteria, each monomer of PSI dimer contains 13 lipid molecules (3:0:4:6) [11]. PSI trimers of *Synechocystis* sp. 6803 include seven asymmetrically distributed SQDG molecules [111]. In PSI tetramer of *Anabaena* sp. PCC 7120, eight SQDGs were found [12], of which four SQDG molecules are located between PsaB and PsaX of all monomers. These four SQDGs play a role in the assembly and stabilization of PsaX in PSI monomer. The side chain of PsaB contacts the head group of one of these SQDGs through two hydrogen bonds. Sulfate group of this SQDG is embedded in a pocket formed by N-terminal helix of PsaX; extensive hydrogen bonds can be formed between the sulfate group and three nitrogens of PsaX backbone and its side chain. Thus, two SQDGs are located within two monomers of a single dimer; in each monomer, SQDG connects PsaX to PsaB. Two more SQDGs are located between the two monomers at the interface I [12].

Those SQDGs in PSI tetramer of *Anabaena* that are located at the interface I are able to form extensive hydrogen bonds with the surrounding residues of two monomers, showing an obvious role in mediating intermonomer interactions. One of the SQDGs is sandwiched between three subunits: two PsaL and one PsaI, and the second one is located between PsaL and PsaB while connected via a hydrogen bond network to N-terminus of PsaL. These lipids play a specific role not only in biogenesis of PSI monomers to stabilize subunit interactions within the monomer, but also in association of monomers into oligomers in tetrameric PSI [12].

The presence of two PG molecules localized at the periphery of PSI core in cyanobacteria implies that PGs may play an important role not only in PSI function, but also in the assembly of PSI core. It has been previously shown that tobacco PSI preparations contain only MGDG and PG in a 2:1 molar ratio, with PG bound to the core of PSI complex. PG has been shown to be required for translation of PSI subunits such as PsaA/B in cyanobacteria. PG deprivation in mutants reduced the functionality of PSI complex but not PSII complex while PG loss inhibited synthesis of PSI complex but not its stability. PG-dependent changes in the molecular organization of both the PSI core and LHCI pigment–protein complexes have also been reported [109].

In contrast to the rapid inhibition of PSII under PG deficiency, PSI is affected only by a longer period (>2 weeks) of PG deficiency. Three PG molecules are integrated into the crystal structure of PSI of *T. elongatus*. One of them is located near the reaction center and the other two are at the periphery. The decreased PSI activity and the increased amount of monomeric PSI complexes after long-term PG deficiency suggest an important role of PG in activity and structural organization of PSI [6,22,103,104].

Thus, PG loss affects the structure of thylakoid membranes and disrupts the RC function of both PSI and PSII. In the core structure of PSI of *T. elongatus*, XRC identified four lipid molecules on the reducing side of PsaA/B polypeptides. Two PG molecules are located at the periphery, and one PG and one MGDG are located near the electron acceptor phylloquinone molecules. In PG-depleted mutant cells of *Synechocystis* sp., the amount of PSI was reduced. At the same time, the amount of PSI trimers and PsaL subunits in them was also reduced, which is consistent with the location of PG in the center of PSI trimers, where PsaL is also present [112]. It should be noted that other phospholipids are sometimes found in PSI. For example, eight phosphatidylethanolamine molecules were found in PSI supercomplex of *Dunaliella salina* [41]. PC molecules were found in biochemical PSI preparations from *Pisum sativum* [95] but they were not shown in the solved X-ray structures of PSI supercomplexes from the plant [33,49,113].

The recent review of Yoshihara and Kobayashi ([20] Table S1) sums up earlier results on the content of lipid molecules in solved XRC and cryoEM structures of PSII, C_2_S_2_M_2_ PSII-LHCII, PSII-FCPII, PSI, PSI-LHCI, Cyt*b_6_f* and NDH. The objects were, respectively: cyanobacteria *T. vulcanus* (three PDB structures), green algae *C. reinhardtii* (three PDB structures), red algae *Cyanidium caldarium*, diatom *Chaetoceros gracilis* (4 PDB structures) and the green plants *Pisum sativum* and *Spinacia oleracea* for PSII complexes; seven cyanobacterial species (nine PDB structures), four species of green algae (seven PDB structures), red algae *Cyanidioschyzon merolae*, diatom *Chaetoceros gracilis* (three PDB structures), the green plants *Pisum sativum* (three PDB structures), *Zea mays* and moss *Physcomitrium patens* for PSI complexes; green plant *Spinacia oleracea* for the Cyt*b_6_f* complex; cyanobacteria *T. vulcanus* (3 PDB structures) for NDH complexes ([20] Table S1). In general, the data presented are quite close to each other within the specified groups. Noticeable differences for PSI-LHI are present for *Pisum sativum* with 19 MGDG molecules in one case instead of typical three–five molecules and for green algae where number of MGDGs is one–three for three cryoEM structures while it makes five–ten molecules for the other four cases ([20] Table S1). The PG content data for PSII-LHCII of *Chlamydomonas reinhardtii* differ, where it varies from 9 to 20 molecules. In green algae, the PG content ranges from eight molecules in *Dunaliella salina* to 24 molecules in *Chlorella ohadii*. These differences could be explained by a number of reasons; here we have species differences and shortcomings in the research methods, when some molecules may remain not visible due to their high mobility. The number of lipid molecules in the photosynthetic complexes of several other objects is given in Table 1.

### 4.5. Involvement of Polar Lipids in the Assembly and Functioning of LHCI and PSI-LHCI

In addition to the main PSI complexes, plants contain LHCI antennae. The results of XRC and cryo-EM analysis of PSI–LHCI and PSI–LHCI–LHCII complexes are summed up earlier [20]. It was found that in PSI-LHCI complexes of *Pisum sativum*, among 10 lipid molecules per monomer (3:1:0:6), five molecules (1:1:0:3) are associated with the main complex [20] while in cryo structure of PSI-LHCI-LHCII of *C. reinhardtii*, 33 lipid molecules (10:2:1:20) were identified, of which 27 are part of PSI-LHCI, and six are part of two LHCII trimers [63]. Lipids characteristic of the PSI core of *C. reinhardtii* are mainly observed at the interface between the LHCI subunits and PSI core and may be necessary for the assembly of PSI-LHCI supercomplex. In particular, the lipid cluster (2:1:1:0) formed around PsaH/I/B may play an important role in the connection with Lhca2-9 antenna in the side layer, which is characteristic only of green algae, while three MGDG molecules located near PsaF and PsaJ can connect Lhca7 and Lhca8 with 2 MGDG in antenna subunits. In addition, a PG molecule is located near Lhca subunit, which is observed in *D. salina* and *C. ohsdii*, and suggests the participation of PG in the coordination of Lhca complex. While MGDG molecule is located symmetrically to PG in the center of PSI core of cyanobacteria, DGDG is included instead of MGDG in algae and plants [20].

In another study, 14 lipid molecules (5:2:0:7) were found in plant PSI-LHCI [14,49]. Four lipids are located in the gap region between PSI core and antennae. Three of them (MGDG, DGDG, and PG) are found between PsaF core subunit and Lhca4 antenna, which may facilitate the binding of Lhca4 to PSI core as linkers during the assembly of this supercomplex [2,33]. Another PG is found at the interface between Lhca1 antenna and the main PsaB/G subunits. DGDG is irregularly located on the stromal side; it is close to B and C helices of Lhca4. The head ring of this DGDG molecule is ideally located between the two benzene rings of PsaF and Lhca4. Another MGDG molecule was found attached to the interface between PsaB and PsaG in the peripheral region of the supercomplex [15]. The region around Lhca1-4 dimer contains the largest number of antenna lipids, indicating a special position of Lhca1-4 dimer that may be vital for the function or assembly of this complex. One of the MGDG molecules close to Lhca4 is distributed at the stromal side, which is an unusual case [14]. In addition to protein subunits and pigments in PSI of the green algae *Dunaliella salina*, 16 lipids (5:2:0:8) including 1 PE were detected (cryo-EM method). Four lipids seem to play a special role in coordination of Chl molecules with their polar head groups [41].

It should be noted that LHC are encoded by nucleus and are transported to chloroplasts after their translation outside the chloroplasts. LHCs contain lipid molecules as structural components and therefore the lipids have to be bound during import into thylakoid membrane or assembly into larger complexes. Five new lipids have been identified in the pea PSI-LHC structure (2.4 Å resolution); most of them are located in the region between Lhca subunits and the core of PSI. These lipid molecules may play an important role in binding Lhca subunits to the core as well as in the assembly of the supercomplex [10,33].

## 5. Cytochrome b_6_f Complex

Cyt b_6_f complex mediates electron transfer from PSII to PSI and generates an electrochemical proton gradient across thylakoid membrane in conjunction with water splitting in PSII [22]. Electron transfer within Cyt b_6_f occurs via quinol (Q) cycle, which catalyzes the oxidation of plastoquinol (PQH_2_) and the reduction in both plastocyanin (PC) and plastoquinone (PQ) at two separate sites via electron bifurcation. The dimeric Cyt b_6_f complex from spinach (cryo-EM at 3.6 Å resolution) contains up to three initially bound PQ molecules. The first, PQ1, is located in one Cyt b_6_f monomer, close to PQ oxidation site (Qp), adjacent to heme b_p_ and Chl *a*. PQ_2_ spans the intermonomer cavity, partially overlapping with the PQ reduction site (Q_n_) at the PQ1 side [96,114]. The heterooligomeric Cyt b_6_f complex, which functions between the RC complexes of oxygenic photosynthetic electron transport chain, contains eight tightly associated polypeptide subunits with a dimer molecular weight of ≈217 kDa [115].

The distribution of Cyt b_6_f complex is controversial, with suggestions that it is entirely localized within the stromal membranes, in a membrane region in contact with the grana and stromal membranes, with the possibility of diffusion into the stromal membranes, or randomly distributed between the granal and stromal regions [94]. The structure of the complex could be found in the set of about 500 heterooligomeric integral membrane proteins solved with a resolution below 2.5 Å [116,117].

Dimeric Cyt b_6_f complex (3.1 Å resolution) from alga *C. reinhardtii* contains 13 TM-α-helices per monomer together with hemes, Chl, β-carotene, and plastoquinone [96,114,115,116,117]. The monomeric form of Cyt b_6_f complex consists of four large subunits: Cyt b_6_, Cyt f, iron-sulfur protein (ISP) and subunit IV, as well as four small subunits—PetG/L/M/N [117]. The TM subunits are based at the stromal side of the complex [96,114,115,116,117].

Crystal structure analysis of Cyt b_6_f from *C. reinhardtii* and two cyanobacteria, *Mastigocladus laminosus* and *Anabaena* sp. PCC 7120, revealed integration of SQDG molecule into this complex [22]. This SQDG molecule is located at the stromal side of the complex and probably serves as a link between TM helices of Cyt f and iron-sulfur protein (ISP). Mutations in the amino acid residues interacting with this SQDG molecule invariably resulted in the disruption of ISP and disruption of the regulation of Cyt f synthesis, indicating the importance of this lipid in formation and organization of Cyt b_6_f complex. In addition, the SQDG molecule forms an intermonomer cavity with two lipid binding sites, which may serve as a channel for quinone/quinol diffusion [20,118].

In the dimer of Cyt b_6_f complex from spinach, 12 selected lipids (2:0:3:4) and 3 PCs have been identified [94,96]. Four of the seven anionic lipid molecules (0:0:2:2) orient their polar head groups toward stroma, whereas all five nonionic lipids orient them toward lumen. MGDG and PC molecules at the edge of the complex are located in gaps of the “picket fence” formed by TM helices of subunit IV and four small subunits (PetG/L/M/N) and may serve as anchors linking these peripheral TM helices to the core and stabilizing the complex. Since PC is absent in thylakoid membrane, the three PCs detected in the structure were derived from molecules exogenously added during purification and crystallization. In the native form of Cyt b_6_f complex in vivo, the sites occupied by these PCs should be occupied by other lipid types, such as DGDG. For example, mass spectroscopic analysis of Cyt b_6_f isolated from spinach detected some DGDG in addition to MGDG, SQDG, and PG [96,118].

Although two non-ionic lipids on the lumenal side of *C. reinhardtii* Cyt b_6_f were previously identified as MGDG, one could be a DGDG molecule based on mass spectroscopic data. It is calculated that 0.3–0.9 mol.% of the total thylakoid lipids are integrated into Cyt b_6_f complexes with a stoichiometry of 1:1:1:1 per monomer. The structure of spinach Cyt b_6_f is expected to contain equal amounts of PG and SQDG. Cyt b_6_f content in cyanobacteria and plants is 5 and 1 mmol/mol Chl, respectively [22].

MGDG and DGDG are known to activate Cyt b_6_f complex and CF0-CF1-ATPase in chloroplasts [94]. Loss of MGDG in a tobacco mutant reduced the levels of Cyt b_6_f complex and blocked electron transfer between PSII and PSI [6,119]. In Cyt b_6_f and NAD(P)H-dehydrogenase-like complexes of the first type, as in photosystems, the stromal/cytosolic sides are negatively charged by PG and SQDG, while their luminal sides are occupied by uncharged galactolipids [10]. The physiological significance of these asymmetric distributions is still unknown.

The structural model of algal Cyt b_6_f contains, in addition to SQDG, two MGDGs located at the lumenal side of membrane. The latter are the examples of interhelical lipids, with MGDG head groups located between adjacent TM helices close to the surface of the protein. Here, the lipids serve as a means of filling gaps in the structure where adjacent TM helices do not run parallel to each other; in usual membranes, this role is realized by sterols [50,120].

In Cyt b_6_f complex of *Masnigocladus laminosus*, there are two interconnected cavities at the dimer interface, each of them appears to facilitate quinone exchange between quinone-binding sites on opposite sides of membrane and in different monomers. Both cavities are presumably filled by two lipid molecules in vitro [51,52]. As a rule, the quinone-binding sites in Cyt b_6_f complexes are usually filled with lipids. Within PSII complex is a Q_B_ binding site in D1 polypeptide [23,89,90]. A cavity has been found that forms a prechamber to plastoquinone reductase (Q_B_) region of the central RC domain. This cavity forms a channel that facilitates the hydrophobic diffusion of plastoquinol from Q_B_ site into membrane, where it is oxidized by Cyt b_6_f [21,60,94]. Four selected lipids (1:2:1:0) are included in PSII structure; they partially fill this cavity [52,121]. Along one side of the cavity is the tail portion of plastoquinone Q_B_ [52]. One of the two PG molecules near Q_A_ has been found to be important for maintaining Q_B_ function and for stabilizing the binding of extrinsic proteins to PSII [105]. The other three PG molecules are located near Q_A_-binding site, the region between CP43 and the D1/D2 heterodimer, and Q_B_-binding site, respectively; however, their functions are unclear. One of them interacts with the α-subunit (PsbE) of Cyt b_559_, which forms a heme-bridged heterodimer with the β-subunit (PsbF) and functions as an essential component of PSII [60].

A transgenic tobacco plant with a 53% reduced MGDG content compared to control plants had a significantly reduced number of thylakoid membranes and growth retardation. It is suggested that MGDG affects not only PSII activity but also Cyt b_6_f, since a tobacco mutant with a reduced MGDG content did not exhibit active electron transfer under illumination, which may be explained by a blockade of electron transport in the Cyt b_6_f complex [92].

## 6. NDH-1 Protein Complex

Electron transfer and reduction in NADP^+^ to NADPH in photosystems creates a proton gradient across the thylakoid membrane for ATP synthesis. In chloroplasts of some plant species and in cyanobacteria NDH or NDH-1 complex (NADH dehydrogenase or type I NADH dehydrogenase) is found; it provides cyclic electron flow around PSI which is linked to several physiological processes in different ways [16,17,18,19]. Complexes like NAD(P)H dehydrogenase type I (NDH-1) are involved in transfer of electrons from ferredoxin or NAD(P)H to plastoquinone pool [20,122]. In cyanobacteria, these complexes have different sizes and subunit isoforms [122]. The NDH-1L complex includes a central membrane domain consisting of 10 subunits (NdhA–C, D1/E/F1/G/L/P/Q) and a peripheral hydrophilic (redox-active) domain consisting of nine subunits (NdhH–K, M–O, S, and V) [97]. The stromal/cytosolic sides of the NAD(P)H dehydrogenase-like complexes of the first type, similar to both photosystems and Cyt b_6_f, are negatively charged by PG and SQDG, while their luminal sides are occupied by uncharged galactolipids [3]. In *T. elongatus* (3.2 Å resolution), the structure of entire NDH-1L complex bound to ferredoxin has been determined; it contains 13 lipid molecules (2:2:3:6) [97] or 15 molecules (0:2:4:9) [19].

The plastoquinone-binding cavity formed by Ndh A/K/H contains two DGDGs, which stabilize this cavity. In space between membrane and hydrophilic domains, two PGs connect N-helix of Ndh I with the membrane domain of Ndh A/C, and one SQDG is located in the cavity formed by N-terminal fragments of Ndh L/N/K. At the Ndh D/B interface, two lipid molecules (SQDG and PG) are also located. At Ndh F1/D1 interface, one MGDG and three PGs [97] or two SQDG and two PGs [19] can be located, which can facilitate the connection of these subunits with Ndh P and NdhQ [20].

In *T. elongatus*, structure of another type of NDH-1, NDH-1MS, was also determined, in which lipid molecules (0:2:5:0) were also identified [123]. This complex includes CO_2_-scavenging proteins (Cup A and Cup S) and has a carboanhydrase activity that hydrates CO_2_ to HCO_3_^−^. There are no lipids at the interface between the Cup A/S module and the membrane domain, and the lipid binding sites in NDH-1MS are similar to those in NDH-1L [20].

Several NDH complexes from higher plants are solved later [124,125]. CryoEM structures of NDH supercomplexes with PSI were determined for *Hordeum vulgare* [124] and for *Arabidopsis thaliana* [125]. NDH from *Hordeum vulgare* had 25 subunits; 24 of them are from four types of subcomplex: SubA/B/M/L with a few lipids detected (0:0:2:1) [124]. NDH from *Arabidopsis thaliana* had 29 subunits of five types SubA/B/E/L/M; 2 PGs and 1 SQDG were determined in the structure of NDH Table S1 in [125]. Recently, the structure of NDH supercomplex with PSI-LHCI from *Spinacia oleracea* was also solved by cryoEM with 3.0–3.3 Å resolution [124,126]. NDH consists of five modules, SubA/B/E/L/M, while the whole supercomplex consists of 41 protein subunits and 192 pigment molecules. The interactions of subunits are reinforced by 46 lipid molecules; 23 lipid molecules are estimated in the NDH membrane arm (5:0:3:14) with PGs most abundant and, surprisingly, no DGDG molecules found [124,126].

In thylakoid membrane, PG or SQDG may be specifically associated with ATP synthase, but no lipid molecules have yet been detected within its structure [20,22]. Proteins anchored to the membrane surface include the ferredoxin-NADPH reductase complex, carboxy dismutase, and the ATP synthase complex. The interaction of these proteins with thylakoid membrane lipids is likely limited to electrostatic interactions with acidic lipids such as PG and SQDG [31]. XRC and cryo-EM methods helped to identify glycerolipid molecules in NDH-1 complexes. It was shown that PG and SQDG increase binding affinity of NDH-1 to plant protochlorophyllide oxidoreductase LPOR, while MGDG affects the spectral properties of the complex and can induce oligomerization [20,121,122,127].

## 7. The Role of Bound Fatty Acids in Photosynthesis

Thylakoid membranes are composed of only four selected classes of lipids, but each of them consists of a number of molecular forms depending on the type of their hydrocarbon chains [128]. Such a diversity of acyl chains and the high content of unsaturated FAs in thylakoid lipids complicate the study of their role in photosynthesis. The function of these membrane systems is closely dependent on fluidity of their lipid matrix, which is influenced by lipid composition and temperature [129]. The acyl chains of lipids interact with this predominantly hydrophobic surface of the protein inside the membrane and are located along the grooves on the protein surface formed between TM helices or resulting from helix rotation [52].

It was found that the influence of unsaturated FAs, which are part of MGDG, is a major factor in phase behavior of this lipid in aqueous systems. When MGDG contain two α-linolenic acid residues, these lipids form a hexagonal structure in water. Removal of *cis*-unsaturated double bonds in MGDG by catalytic hydrogenation converts the structure into a bilayer [31]. In addition to polar head groups, the FA chains also determine the physicochemical properties of glycerolipids. It is possible that FAs of newly synthesized glycerolipids are unsaturated in membranes, and then the new molecular forms of glycerolipids are incorporated into photosynthetic proteins during the assembly of photosynthetic complexes [10]. The double bonds of FAs of thylakoid membranes are usually in *cis* position and very rarely in *trans* position, as in *trans*16:1^3^. However, the importance of this FA for photosynthesis is undoubtedly great and has not yet been explained.

Desaturase FAD7 produces polyunsaturated FAs. It should be noted that thylakoid membranes and PSII complex of *T. vulcanus* and *T. elongatus* have only 16:0, 16:1, 18:0, and 18:1 FAs in their composition and do not have polyunsaturated acids [86]. FAD7 activity levels modulate the relative abundance of triene and diene FAs, which are contained in lipids of other membranes. Their ratio, as well as the ratio of saturated/unsaturated FAs, affects the physical properties of lipids and, thus, the structure and function of membrane [130]. For example, high levels of desaturation decrease the melting temperature of lipids and increase membrane fluidity, facilitating lateral diffusion of lipids, proteins, and other molecules within the membrane [99]. Thus, due to its influence on properties of membranes, FAD7 activity can potentially influence photosynthesis [130]. Regardless of the genotype of higher plants and algae, α-linolenic FA (18:3^Δ9,12,15^) always dominates in chloroplast lipids, where its level in galactolipids of thylakoid membranes reaches 90% or more. It can be argued that α-linolenic FA plays a global role on our planet. Why, of all plant FA, is this acid predominant in photosynthetic apparatus and plays an important structural and metabolic role? A rigorous model experiment was conducted to study the ability of methyl esters of α-linolenic acid and other FAs of plant tissues to transition to an excited state; in a chamber with strontium-90 and argon at 190° C. In this case, ionization intensity of esters of α-linolenic acid with an increase in voltage in the chamber from 750 to 1500 V increased by 60%, in contrast to esters of other FAs [131]. Whether this increased ability of α-linolenic acid to transition to an excited state is related to process of photosynthesis is still unknown. Considering the structural features of this FA, it can be seen that at the methyl end of its chain there is an uncharged system of three double bonds, which are separated from the methyl group by three, six, and nine carbon atoms, respectively. A similar system of double bonds is also contained in the chain 16:3^Δ7,10,13^, which, very importantly, is found only in chloroplasts and differs from linolenate by the absence of two methylene groups at the carboxyl. Unfortunately, this FA was absent in the experiment with transition to an excited state [131].

**Figure 3:**
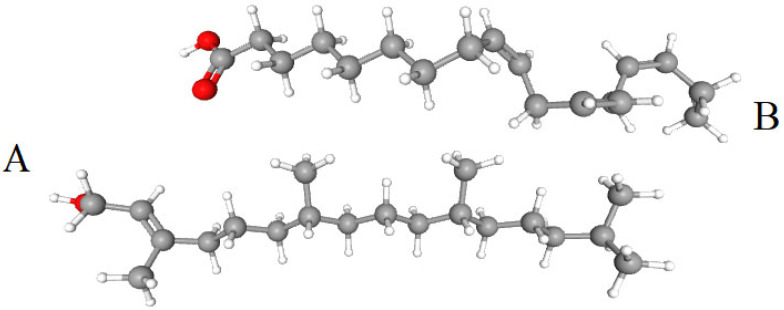
Chlorophyll residue phytol with its methyl groups, which structurally fit to the system of double bonds of α-linolenic acid. (**A**) Phytol; (**B**) α-linolenic acid. The corresponding 3D conformers of the molecules are chosen from PubChem (https://pubchem.ncbi.nlm.nih.gov/compound/5280435 (accessed on 15 July 2025), rotated conformer 2, and https://pubchem.ncbi.nlm.nih.gov/compound/5280934 (accessed on 15 July 2025), rotated conformer 2).

The significance of this ω-3,6,9-system of double bonds of triene FAs in plastid thylakoids is that it is capable of adopting a helical conformation. In this case, a close association is formed with helical chains of membrane proteins of plastid lamellae due to hydrophobic interaction of this system with π-orbitals of aromatic amino acid residues [130]. In addition, this system is complementary in structure to phytol residue of Chl, where the 7-, 11-, and 15-methyl groups of phytol fill pockets in the α-linolenate configuration at the sites of its *cis*-double bonds (Figure 3). Such complexes of galactolipids with proteins and pigments provide an optimal spatial orientation of hydrophilic photoreceptor porphyrin structures of Chl at the surface of lipoprotein membranes and enable unimpeded electron transfer in an anhydrous medium with a low dielectric constant. At the same time, the increased ability of α-linolenic FA to be oxidized can hardly be the reason for its “leadership” in chloroplasts, since in this respect 18:3 differs little from 18:2.

With increased activity of chloroplast PSI, active ω3-desaturation of FA occurs. This introduces an additional double bond into the precursor molecule, usually FA of ω6 series, which esterifies the predominant thylakoid membrane lipids—MGDG. This ω3-desaturation is important in optimizing the light regime, leading to activation of PSI in the process of formation of chloroplast photosystems in cell cycle. The reaction of ω3-desaturation of FA has been shown to correlate with the presence of PSI. It is assumed that ω3-desaturation takes place in the complex of primary processes of redistribution and/or discharge of light energy and in oxidation-reduction reactions of chloroplasts [132].

Finally, PSII lipids contribute to the binding sites of Chl molecules and carotenoids associated with RC of PSII [23,61,90]. Hydrophobic interactions between lipid FAs and Chl phytol chains, as well as interactions between lipid head groups and chlorin rings of Chl *a* molecules have been described for interactions with Chl molecules [21,50]. Lipid-carotenoid interactions are less specific, and hydrophobic amino acids of proteins and lipid FAs are often involved in the creation of a specific carotenoid binding site [61].

It should be noted that SQDG contains an increased content of palmitic acid (16:0). For example, in green alga *Ulva fenestrata*, MGDG contains only 1% of it, while SQDG contains 30%. In *Chlorella pirenoidosa*, the content of 16:0 in MGDG and SQDG is 3% and 67%, respectively. It is worth noting that such phospholipids as PI are also rich in this 16:0 FA [133]. The role of this FA in photosynthesis processes has been studied very little so far. It has been shown that exogenous addition of free FAs affects photoinhibition of PSII in *Synechocystis* sp. Moreover, such saturated FA as 16:0, 18:0 and their analogs with a longer chain promoted recovery of PSII, accelerating de novo synthesis of D1 and reducing photoinhibition of PSII [107]. *Synechocystis* sp. PCC6803 cells were exposed to bright light, which caused photoinhibition. However, exogenous addition of free FA such as 16:0, 18:0 and longer-chain saturated FA to these cells enhanced PSII reduction by accelerating de novo D1 synthesis and thereby reducing photoinhibition [109].

Of considerable interest is that chloroplast FAs include a unique *trans*-16:1^3^ (up to 3.5% of total FAs). Moreover, it is almost entirely concentrated in sn-2 position of glycerol of PG [130,134]. It should be noted that the sn-1 position in PG is occupied mainly by either 16:0 or 18:3 FAs, which differ greatly in their properties. They also compete with trans-16:1^3^ for a place in the sn-2 position. Thus, the content of trans-16:1^3^, 16:0 and 18:3, which are the main ones in PG of thylakoid membranes of tobacco plants, is 31, 29, and 23 mol.%, respectively [135]. Is the presence of *trans*-16:1^3^ in PG makes one of the reasons for its selection for photosynthesis? The concentration of this FA in chlorella increases almost 2-fold with increasing light intensity, which reflects the reorganization of chloroplast lipids under the influence of light [132]. It is possible that it is this FA that allows PGs to fill certain spaces in photosystems where PG is represented at a higher level compared to its total content in thylakoid membranes [136,137].

It has been shown that the primary and secondary products of non-enzymatic and enzymatic lipid peroxidation (LPO) have the ability to modify PSII proteins. LPO products formed under light and heat stress in thylakoid membranes cause oxidative modification of proteins in PSII of higher plants. Damage to PSII proteins by LPO products is the mechanism underlying photoinhibition and heat inactivation [137]. Under light stress, illumination-induced LPO is involved in the damage of D1 protein and LHCII subunits. At the same time, artificial addition of lipoxygenase in the dark caused inhibition of PSII activity in thylakoids, production of singlet oxygen, and damage to D1 protein similar to that occurring with LPO [138]. It is suggested that singlet oxygen formed as a result of LPO under light stress is mainly involved in the damage of PSII subunits [134,137].

## 8. Conclusions

Polar lipids create a matrix in the form of membranes for the formation and existence of complexes PSII, PSI, Cyt b_6_f, and NADP. However, it is obvious now that the role of polar lipids is essentially larger than being a simple lipid bilayer. Special groups of these lipids are directly included in the composition of these complexes: without them photosynthesis cannot be carried out by functioning of proteins and pigments alone. The number and location of these lipids within individual complexes of PSII and PSI and between them, as well as in Cyt b_6_f and NADP complexes, have not yet been determined clearly. More detailed information about the lipid binding sites in PSII–LHCII and PSI–LHCI supercomplexes can also serve as a basis for discovering the role of these lipids. The functional role of lipids in general, as well as independent role of their polar head groups or chains of their associated FA in the processes of photosynthesis themselves, has also not yet been sufficiently studied and requires additional research. This includes the role of galactopyranosyl, sulfoquinovose, glycerol, and phosphate (in PGs) rings of the lipid head groups and a number of FAs (18:3^Δ9,12,15^, 16:3^Δ7,10,13^, *trans*-16:1^3^ and 16:0) in the structure of photosystem complexes.

It can be seen that the data available in the literature on the content of individual lipid molecules in photosystems and their supercomplexes, as well as on the locations of these molecules, are still few in number, incomplete, and often differ from one author to another. Therefore, here we have a huge field for future research. Information about the role of lipids in the structural and functional dynamics of photosynthetic complexes is accumulating. Close interactions have been found between multicomponent protein–cofactor complexes and membrane lipids. However, the relationship between photosynthetic activity, the conformational and functional state of thylakoid membrane proteins and their lipid composition, which can change under different environmental conditions, remains a subject of future research.

The information collected by X-ray crystallography provides new opportunities for search of molecular explanations for the observed effects of different lipid types on the structure, mechanism of functioning and organization of RCs and other photosynthetic proteins. Recent advances in cryo-EM have contributed to a leap forward in structural biology; macromolecular structures are no longer rely on X-ray crystallography only. Although X-ray crystallography plays an important role in high-resolution (<3 Å) structure determination, cryo-EM has the advantage of characterizing less stable complexes in a near-natural environment; thus, a combination of both techniques is important. These techniques can be complemented by computational approaches using molecular dynamics (MD) modeling, which allows the study of conformational changes in membrane proteins in membranes with defined lipid content. Computer 3D MD modeling using supercomputers and computer technologies will likely reveal the place and functional role of each lipid molecule, as well as all protein molecules, Chl, carotenoids, quinones, and other cofactors in the photosystems and cytochrome complexes.

## Data Availability

Data are contained within the manuscript.

## Abbreviations

Chl	chlorophyll
Cyt b_6_f	cytochrome b_6_f
Cryo-EM	single-particle cryo-electron microscopy
DPG	diphosphatidylglycerols (cardiolipins)
FAs	fatty acids
ISP	iron-sulfur protein
LHCII and LHCI	major light-harvesting complexes II and I
LPO	lipids peroxide oxidation
MGDG and DGDG	mono- and digalactosyldiacylglycerols
NADP^+^ and NADPH	nicotinamide adenine dinucleotide phosphate in oxidized and reduced forms
PCs	phosphatidylcholines
PEs	phosphatidylethanolamines
PG	phosphatidylglycerols
PL	polar lipids
PSII and PSI	photosystems II and I
PQ (Q_A_ and Q_B_)	plastoquinone electron acceptors (primary and secondary)
RC	reaction centers of photosystems
XRC	Xray crystallography
SQDG	sulfoquinovosyl diacylglycerols
TM	transmembrane (proteins or helices)
